# Personalized, autologous neoantigen-specific T cell therapy in metastatic melanoma: a phase 1 trial

**DOI:** 10.1038/s41591-024-03418-4

**Published:** 2025-01-03

**Authors:** Jessica S. W. Borgers, Divya Lenkala, Victoria Kohler, Emily K. Jackson, Matthijs D. Linssen, Sebastian Hymson, Brian McCarthy, Elizabeth O’Reilly Cosgrove, Kristen N. Balogh, Ekaterina Esaulova, Kimberly Starr, Yvonne Ware, Sebastian Klobuch, Tracey Sciuto, Xi Chen, Gauri Mahimkar, Joong Hyuk F. Sheen, Suchitra Ramesh, Sofie Wilgenhof, Johannes V. van Thienen, Karina C. Scheiner, Inge Jedema, Michael Rooney, Jesse Z. Dong, John R. Srouji, Vikram R. Juneja, Christina M. Arieta, Bastiaan Nuijen, Claudia Gottstein, Olivia C. Finney, Kelledy Manson, Cynthia M. Nijenhuis, Richard B. Gaynor, Mark DeMario, John B. Haanen, Marit M. van Buuren

**Affiliations:** 1 Department of Medical Oncology, Netherlands Cancer Institute (NKI), Amsterdam, The Netherlands; 2 BioNTech US, Cambridge, MA USA; 3 BioTherapeutics Unit, Division of Pharmacy and Pharmacology, Netherlands Cancer Institute (NKI), Amsterdam, The Netherlands; 4 BioNTech SE, Mainz, Germany; 5 Division of Molecular Oncology and Immunology, Netherlands Cancer Institute (NKI), Amsterdam, The Netherlands

**Keywords:** Drug development, Tumour immunology

## Abstract

New treatment approaches are warranted for patients with advanced melanoma refractory to immune checkpoint blockade (ICB) or BRAF-targeted therapy. We designed BNT221, a personalized, neoantigen-specific autologous T cell product derived from peripheral blood, and tested this in a 3 + 3 dose-finding study with two dose levels (DLs) in patients with locally advanced or metastatic melanoma, disease progression after ICB, measurable disease (Response Evaluation Criteria in Solid Tumors version 1.1) and, where appropriate, BRAF-targeted therapy. Primary and secondary objectives were evaluation of safety, highest tolerated dose and anti-tumor activity. We report here the non-pre-specified, final results of the completed monotherapy arm consisting of nine patients: three at DL1 (1 × 10^8^–1 × 10^9^ cells) and six at DL2 (2 × 10^9^–1 × 10^10^ cells). Drug products (DPs) were generated for all enrolled patients. BNT221 was well tolerated across both DLs, with no dose-limiting toxicities of grade 3 or higher attributed to the T cell product observed. Specifically, no cytokine release, immune effector cell-associated neurotoxicity or macrophage activation syndromes were reported. A dose of 5.0 × 10^8^–1.0 × 10^10^ cells was identified for further study conduct. Six patients showed stable disease as best overall response, and tumor reductions (≤20%) were reported for four of these patients. In exploratory analyses, multiple mutant-specific CD4^+^ and CD8^+^ T cell responses were generated in each DP. These were cytotoxic, polyfunctional and expressed T cell receptors with broad functional avidities. Neoantigen-specific clonotypes were detected after treatment in blood and tumor. Our results provide key insights into this neoantigen-specific adoptive T cell therapy and demonstrate proof of concept for this new therapeutic approach. ClinicalTrials.gov registration: NCT04625205.

## Abstract (web summary)

In this phase 1 trial of a personalized, neoantigen-specific autologous T cell therapy, BNT221, when given as monotherapy in patients with metastatic melanoma refractory to PD-1 and CTLA-4 inhibitor regimens, the therapy was safe and showed preliminary clinical activity and neoantigen-specific T cell responses.

## Main

Immune checkpoint blockade (ICB) or, more specifically, inhibition of programmed cell death protein 1 (PD-1) and cytotoxic T lymphocyte-associated protein 4 (CTLA-4), has become standard first-line and second-line treatment in advanced unresectable melanoma^1,2^. ICB treatment can result in durable responses in 45% of patients treated with anti-PD-1 monotherapy and in up to 58% of patients treated in combination with anti-CTLA-4 (ref. ^3^). However, a significant proportion of the patients do not respond to ICB or develop resistance^3–5^. Targeted therapies are available for patients with melanomas expressing *BRAF* mutations; however, responses are not durable for most patients^4^. Despite these considerable advances, patients with advanced melanoma whose disease does not respond or becomes refractory to current standard-of-care treatments have very limited treatment options, and additional treatment approaches are warranted for this patient population.

Adoptive T cell therapies (ACTs) hold promise to fulfill this objective. Tumor-infiltrating lymphocytes (TILs) and engineered T cell receptor-T cells (TCR-Ts) have shown encouraging results for solid tumors^6,7^. However, there are several challenges, including identification of suitable targets, overcoming the immunosuppressive tumor microenvironment (TME) and ensuring sufficient persistence in vivo. An important advance has been a shift from bona fide cancer/testis antigens to exploring neoantigens—antigens derived from non-synonymous cancer mutations. Their immunogenicity and tumor specificity make them ideal targets for cancer therapy, and proof of concept of this has been demonstrated in the context of neoantigen-targeting vaccines^8–12^. Targeting neoantigens warrants a fully personalized therapeutic approach, given that the large majority of neoantigens are unique to each patient’s tumor.

Thus far, most ACT successes for advanced melanoma have been observed with TIL treatment: favorable phase 2 and 3 clinical trial results have been reported^13–15^, and Lifileucel is now approved by the US Food & Drug Administration (FDA) for this indication^16^. For this approach, tumor-infiltrating T cells are harvested via surgical resection and expanded during in vitro culture. After a lymphodepleting chemotherapy regimen, patients receive their TIL product. However, not all patients have a surgically accessible tumor or sufficient lymphocyte infiltration in their tumor to obtain the required number of cells for manufacturing. Furthermore, the immunosuppressive nature of the TME may lead to T cells with a range of functional states, with some becoming irreversibly dysfunctional^17–19^. Optimizations are under development, such as selecting for the tumor-reactive or, more specifically, the neoantigen-specific TIL among the bulk product^20,21^.

In the TCR-T approach, T cells are obtained from peripheral blood via leukapheresis, which has the potential to acquire T cells in a less dysfunctional state^19^. They are then genetically modified to express specific TCRs, which recognize tumor antigens, such as MART-1, MAGE-A4 or NY-ESO-1 (refs. ^22–25^), or neoantigens^26–30^. However, TCR-Ts are usually restricted to one particular epitope, presented by a single human leukocyte antigen (HLA) subtype, thereby limiting the number of patients eligible for such therapies and increasing the risk of antigen escape^26,31–33^.

BNT221 aims to address these limitations of prior cell therapies. This new class of ACT combines the precision of a neoantigen approach with highly functional T cells derived from peripheral blood. The result is a drug product (DP) with broad TCR diversity targeting multiple patient-specific neoantigens. BNT221 is generated through an ex vivo induction process (NEO-STIM) that primes, activates and expands both CD8^+^ and CD4^+^ T cell responses against neoantigen epitopes without genetic manipulation. Before NEO-STIM, patient-specific mutations are identified through whole-exome sequencing (WES), followed by a bioinformatics approach to identify neoantigen epitopes that are expressed and presented on either major histocompatibility complex (MHC) class I or class II. Subsequently, the DPs are manufactured and infused back into the patient.

We showed previously that BNT221 T cell products can be generated, which are polyclonal, functional and able to kill antigen-expressing cells^34^. We now report clinical safety, activity and translational results after the successful completion of the BNT221 monotherapy arm of the first-in-human (FIH) trial NCT04625205.

## Results

### Successful manufacturing of the T cell DPs

**Fig. 1:**
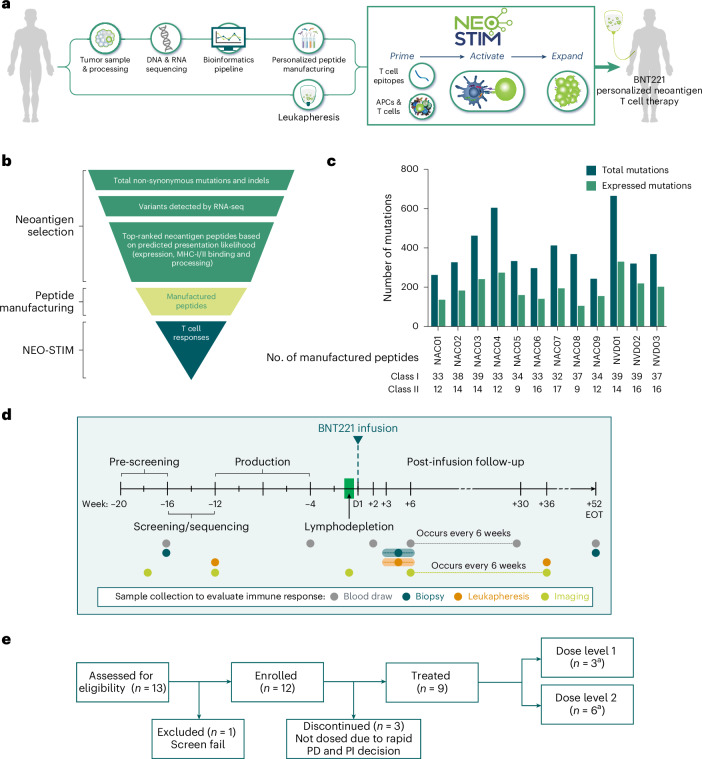
Patient enrollment, manufacturing process and trial design. **a**, NEO-STIM manufacturing process. A tumor and reference blood sample from each patient underwent WES; the tumor sample was also analyzed by RNA-seq, and personalized neoantigens were predicted. Synthetic peptides covering the epitopes were synthesized. These peptides were used in the cell culture process (NEO-STIM) to prime, activate and expand T cells isolated from the patient’s leukapheresis product to generate the BNT221 DP, which was subsequently infused back into the patient after a lymphodepleting chemotherapy regimen. The figure panel was created in BioRender (Gottstein, C. https://BioRender.com/z33v290 (2024)). **b**, Epitope flow from initial sequencing results to the final T cell response. Variants detected by RNA-seq in the tumor samples were considered to be expressed. The expression of the mutation was calculated as a percentage of reads with the mutation multiplied by gene’s TPM and FPKM to remove gene length bias. Peptides representing 40 top-ranked short epitopes (MHC class I) and 20 top-ranked long epitopes (MHC class II or I/II) were selected for manufacturing. **c**, Quantification of total and expressed mutations as well as number of manufactured peptides out of the 40 selected class I and 20 selected class II peptides. Abbreviations for patient IDs: NAC, neoantigen cell dose received; NVD, never dosed. **d**, NCT04625205 trial schematic. **e**, Patient enrollment: out of 13 patients, 12 initially fulfilled eligibility criteria, but three of those were not dosed due to rapid PD and principal investigator (PI) decision. ^a^Of the nine treated patients, three were assigned to DL1 and six to DL2. The disposition schematic reflects the intent-to-treat population. Actual received doses were lower for three patients in DL2 (see ‘Identification of highest tolerable dose’ subsection and Extended Data Table 2). D, day; FPKM, fragments per kilobase of transcript per million mapped reads; TPM, transcripts per million.

The personalized manufacturing process was initiated by identifying targetable neoantigens through WES, RNA sequencing (RNA-seq) and computational methods to predict MHC binding^35–37^. Subsequently, peptides encoding these neoantigens were used for the ex vivo induction process, NEO-STIM, to prime, activate and expand neoantigen-specific T cells. The final DP was then infused into the patient after a lymphodepleting chemotherapy regimen (Fig. 1a,d).

Patients’ tumor tissue as well as peripheral blood mononuclear cells (PBMCs) as a reference were analyzed by WES and RNA-seq to assess mutational burden and quantify the RNA expression level for each mutation. The number of non-synonymous mutations and insertions and deletions (indels) across all patients ranged from 243 to 665 (median 351), and 50.7% of them were expressed (median, range 28.5–68.4%; Fig. 1b,c). This was in line with previous data for melanoma^38^ and provided a good starting point to select suitable targets. Neoantigens with the desired qualities (including high RNA expression, predicted binding to patient-specific MHC molecules and predicted proteasome processing) were then selected^35,36^.

For each patient, 40 short peptides (8–12 amino acids (aa), predicted to bind to MHC class I) and 20 long peptides (25 aa, predicted to bind to MHC class II or both I and II) were selected. On average, 35 short and 13 long peptides (ranges 32–39 and 9–17, respectively) were manufactured (Fig. 1b,c).

**Extended Data Fig. 1:**
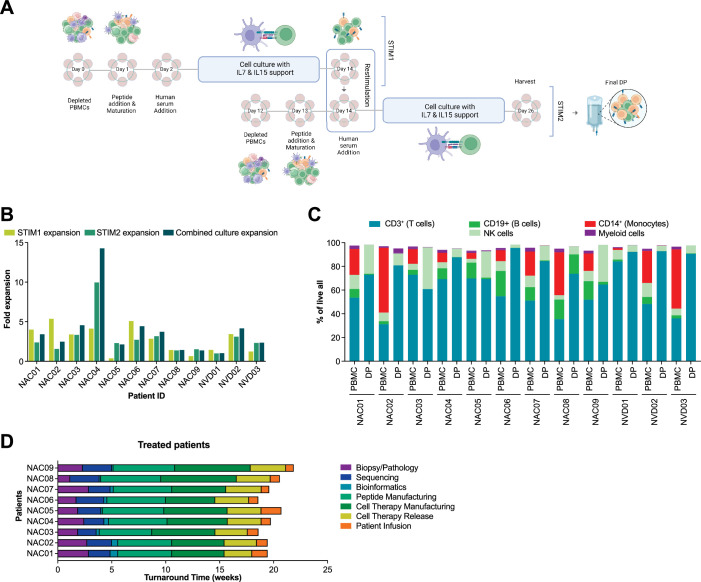
NEO-STIM manufacturing process is feasible and reproducible. **a**. Manufacturing process. STIM1: First set of cell culture vessels, which went through the NEO-STIM process for 14 days; note that up to six vessels (depicted as six circles arranged in a bigger circle) were cultured in parallel; STIM2: Second set of cell culture vessels, which were thawed 12 days later than STIM1 and went through NEO-STIM; STIM1 and STIM2 were combined on day 14. The combined cell culture was then expanded until day 26, harvested, and formulated. An aliquot was removed for quality control, and the remaining formulated T cells were frozen until infusion. Abbreviations: DP: drug product; IL7: interleukin-7; IL15: interleukin-15; PBMC: peripheral blood mononuclear cells (from patient leukapheresis). The figure panel was created in BioRender (Gottstein, C. https://BioRender.com/m09o437 (2024)). **b**. Expansion of cells measured separately for first round of stimulation (STIM1; [Cells Harvested D14] / [Cells Seeded D0]), second round of stimulation (STIM2; [Cells Harvested D26] / [Cells Seeded D12 + Cells Restimulated D14]) and for the combined cell culture ([Cells Harvested D26] / [Cells Seeded D0 + Cells Seeded D12]). **c**. Distribution of the different blood cell subtypes in PBMC (pre-NEO-STIM) and in the final DP in all patients. **d**. Turnaround times from tumor biopsy to release of product, including infusion to the patient (needle-to-needle time) for DPs of all treated patients. The cell therapy manufacturing step contains transfer time between peptide manufacturing and start of cell therapy manufacturing, the latter always being 26 days. Abbreviations for patient IDs: NAC: neoantigen cell dose received; NVD: never dosed.

DPs were manufactured for 12 enrolled patients, nine of whom received the product. A polyclonal T cell product targeting multiple epitopes was generated by initiating six parallel culture vessels containing antigen-presenting cells (APCs), autologous T cells and up to 10 peptides per vessel to prime neoantigen-specific T cells. To further expand already primed neoantigen-specific T cells and provide an additional opportunity to prime these cells, a second culture was initiated on day 12 and was used for restimulation of the first culture on day 14. All cultures were pooled and harvested on day 26 (Extended Data Fig. 1a). The overall expansion during ex vivo culture was, on average, 3.8-fold (median 3.0, range 1.0–13.2; Extended Data Fig. 1b), and the final DP consisted of 80.3% CD3^+^ cells on average (median 82.5%, range 60.7–95.4%; Extended Data Fig. 1c). The procedure to manufacture the DP, from biopsy to infusion (needle-to-needle turnaround time) took 19.8 weeks on average (range 18.5–21.5 weeks) for all treated patients (Extended Data Fig. 1d).

### Clinical trial design and baseline patient characteristics

NCT04625205 is an ongoing, open label, FIH phase 1 clinical study to evaluate BNT221 in patients with unresectable or metastatic melanoma refractory to ICB. Primary objectives are safety and determination of the highest tolerable dose. The secondary objective is clinical efficacy. Exploratory objectives include characterization of the DP and of the neoantigen-specific T cells from patients’ blood and tumor, both pre-infusion and post-infusion. A complete summary of the clinical study design can be found in the Methods section. The study schedule, including pre-treatment lymphodepletion chemotherapy, BNT221 infusion and sample collections to evaluate these endpoints, is provided in Fig. 1d.

For the dose-finding monotherapy arm, patients were enrolled at the Netherlands Cancer Institute (NKI) between December 2020 and October 2022. Of the 13 patients assessed for eligibility, 12 were enrolled; three were not dosed due to progressive disease (PD); and nine received BNT221. Three of the nine patients were assigned to BNT221 dose level (DL) 1 and six patients to DL2 (Fig. 1e).

All patients were heavily pre-treated, with an average of three prior lines of therapy for their metastatic disease (Extended Data Table 1). In addition to PD-1 inhibitor and CTLA-4 inhibitor, two patients received BRAF/MEK-directed therapy. A subset of patients (5/9) also received melanoma-directed bridging therapy during the BNT221 production phase of the study, based on the clinical discretion of the investigator and upon sponsor approval. In such cases, bridging therapy was discontinued before treatment with BNT221 (Extended Data Table 2).

### Safety profile of BNT221

The patients were closely monitored for adverse events (AEs) over the course of the study. We defined treatment-emergent adverse events (TEAEs) as AEs occurring on or after the administration of BNT221 and related to trial treatment. All nine patients experienced at least one TEAE (Table 1). The most commonly reported related TEAEs (occurring in ≥20% of patients) were neutrophil count decreased (100%), white blood cell count decreased (100%), lymphocyte count decreased (100%), anemia (78%), alopecia (56%), fatigue (44%), pyrexia (33%), dysgeusia (33%), platelet count decreased (22%), back pain (22%), device-related infection (33%) and maculo-papular rash (22%). None of the TEAEs was higher than grade 2, with the exception of hematologic AEs. The latter are anticipated as a result of the lymphodepletion regimen^39^ and, therefore, likely not attributable to BNT221. High-grade neutropenia was well managed with daily filgrastim support initially after BNT221 infusion, per study design. No cytokine release syndrome (CRS), immune effector cell-associated neurotoxicity syndrome (ICANS) or macrophage activation syndrome (MAS) was observed. No dose-limiting toxicities or any related serious AEs (SAEs) were reported. A complete list of all study-related AEs, including those before BNT221 treatment, is provided in Supplementary Table 1.

**Table 1:** BNT221-related TEAEs

System organ class and preferred term	DL1 (*n* = 3) *n* (%)	DL2 (*n* = 6) *n* (%)	Total (*n* = 9) *n* (%)
**All grades**			
**Any related TEAE**	**3 (100)**	**6 (100)**	**9 (100)**
**Blood and lymphatic disorders**	**2 (67)**	**5 (83)**	**7 (78)**
Anemia	2 (67)	5 (83)	7 (78)
**Cardiac disorders**	**1 (33)**	**0 (0)**	**1 (11)**
Conduction disorders	1 (33)	0 (0)	1 (11)
**Gastrointestinal disorders**	**1 (33)**	**2 (33)**	**3 (33)**
Constipation	1 (33)	0 (0)	1 (11)
Diarrhea	0 (0)	1 (17)	1 (11)
Nausea	0 (0)	1 (17)	1 (11)
**General disorders and administration site conditions**	**2 (67)**	**4 (67)**	**6 (67)**
Fatigue	1 (33)	3 (50)	4 (44)
Pyrexia	1 (33)	2 (33)	3 (33)
**Infections and infestations**	**1 (33)**	**3 (50)**	**4 (44)**
Device-related infection	0 (0)	3 (50)	3 (33)
Skin infection	1 (33)	0 (0)	1 (11)
**Investigations**	**3 (100)**	**6 (100)**	**9 (100)**
Blood creatinine increased	0 (0)	1 (17)	1 (11)
Blood lactate dehydrogenase increased	1 (33)	0 (0)	1 (11)
Lymphocyte count decreased	3 (100)	6 (100)	9 (100)
Neutrophil count decreased	3 (100)	6 (100)	9 (100)
Platelet count decreased	1 (33)	1 (17)	2 (22)
White blood cell count decreased	3 (100)	6 (100)	9 (100)
**Musculoskeletal and connective tissue disorders**	**2 (67)**	**0 (0)**	**2 (22)**
Back pain	2 (67)	0 (0)	2 (22)
**Nervous system disorders**	**1 (33)**	**3 (50)**	**4 (44)**
Dysgeusia	1 (33)	2 (33)	3 (33)
Headache	0 (0)	1 (17)	1 (11)
Paresthesia	1 (33)	0 (0)	1 (11)
**Respiratory, thoracic and mediastinal disorders**	**1 (33)**	**0 (0)**	**1 (11)**
Cough	1 (33)	0 (0)	1 (11)
**Skin and subcutaneous tissue disorders**	**3 (100)**	**4 (67)**	**7 (78)**
Alopecia	1 (33)	4 (67)	5 (56)
Erythema	1 (33)	0 (0)	1 (11)
Pruritus	1 (33)	0 (0)	1 (11)
Rash maculo-papular	0 (0)	2 (33)	2 (22)
Scar pain	1 (33)	0 (0)	1 (11)
**Vascular disorders**	**0 (0)**	**1 (17)**	**1 (11)**
Flushing	0 (0)	1 (17)	1 (11)
**≥ Grade 3**			
**Any related TEAE ≥ grade 3**	**3 (100)**	**6 (100)**	**9 (100)**
**Blood and lymphatic disorders**	**0 (0)**	**1 (17)**	**1 (11)**
Anemia	0 (0)	1 (17)	1 (11)
**Investigations**	**3 (100)**	**6 (100)**	**9 (100)**
Lymphocyte count decreased	3 (100)	5 (83)	8 (89)
Neutrophil count decreased	3 (100)	6 (100)	9 (100)
White blood cell count decreased	2 (67)	6 (100)	8 (89)

BNT221-related TEAEs by system organ class term (bold), preferred term, incidence and grade. TEAEs were defined as any AE occurring during or after the administration of BNT221 and related to trial treatment. Assignment to DLs per intent to treat. See Extended Data Table 2 for actual doses received. Note that headers are also in bold.

### Identification of highest tolerable dose

To identify the highest tolerable dose, a 3 + 3 dose-finding study was carried out with two DLs: DL1 (1 × 10^8^–1 × 10^9^ total cells) and DL2 (2 × 10^9^–1 × 10^10^ total cells). The first three patients were assigned to DL1 followed by six to DL2 (Extended Data Table 2). Despite the DP cell count being out of specification (OOS) for three patients in DL2, these patients were nevertheless treated, as the total number of cells was either within the range of DL1 or between DL1 and DL2.

The dose for further investigation was identified as 5.0 × 10^8^–1.0 × 10^10^ cells, based on collective data for safety, activity signals and manufacturing feasibility. The observed safety profile supported the highest dose of this range while activity signals were also observed at lower doses; therefore, the broader cell dose range was selected for further clinical study.

### Anti-tumor activity

**Fig. 2:**
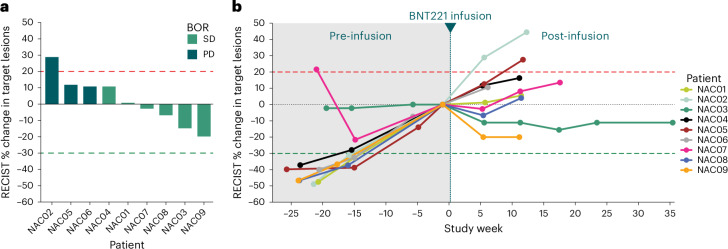
Clinical efficacy. **a**, Waterfall plot of clinical responses based on RECIST 1.1. Baseline measurement is the CT scan taken on day −7 before the BNT221 infusion. For patients NAC05 and NAC06, RECIST was reported as PD due to the emergence of non-target lesions. **b**, Spider plot of clinical responses. Patient NAC07 had a resection of one of the target lesions before infusion, which accounted for the change in sum of target lesions at week −15. Note: five patients received bridging therapy during the manufacturing period before BNT221 dosing; these were patients NAC01, NAC03, NAC05, NAC06 and NAC09. No patient had a response reported from these salvage regimens. Treatments were approved therapies in all cases, and details are provided in Extended Data Table 2. Abbreviations for patient IDs: NAC, neoantigen cell dose received; NVD, never dosed.

BNT221 anti-tumor activity was assessed as a secondary endpoint for the study. Of the nine treated patients, six had stable disease (SD) as their best overall response, including up to 36 weeks for patient NAC03 (NAC, neoantigen cell dose received; Fig. 2a,b). Four of these patients with SD showed tumor regression (up to −20% according to Response Evaluation Criteria in Solid Tumors (RECIST) version 1.1) after BNT221 infusion (NAC03, NAC07, NAC08 and NAC09). Notably, three of these four patients had experienced significant tumor progression in the weeks leading up to their infusion (Fig. 2b). Two patients (NAC08 and NAC09) had self-reported improvements in disease-related symptoms, including improved range of motion, reduced edema in affected extremities and enhanced ability to perform basic activities of daily living. Furthermore, circulating tumor DNA (ctDNA) was evaluated as an additional data source to monitor tumor kinetics. These data largely mirrored trends seen in the tumor measurement data (Supplementary Fig. 1).

Additional planned response secondary endpoints not reported in this paper include duration of response (DOR), clinical benefit rate (CBR), time to first subsequent therapy, progression-free survival (PFS) and overall survival (OS).

### Exploratory outcomes

#### Quantification of polyclonal CD8^+^ and CD4^+^ responses in DP

**Fig. 3:**
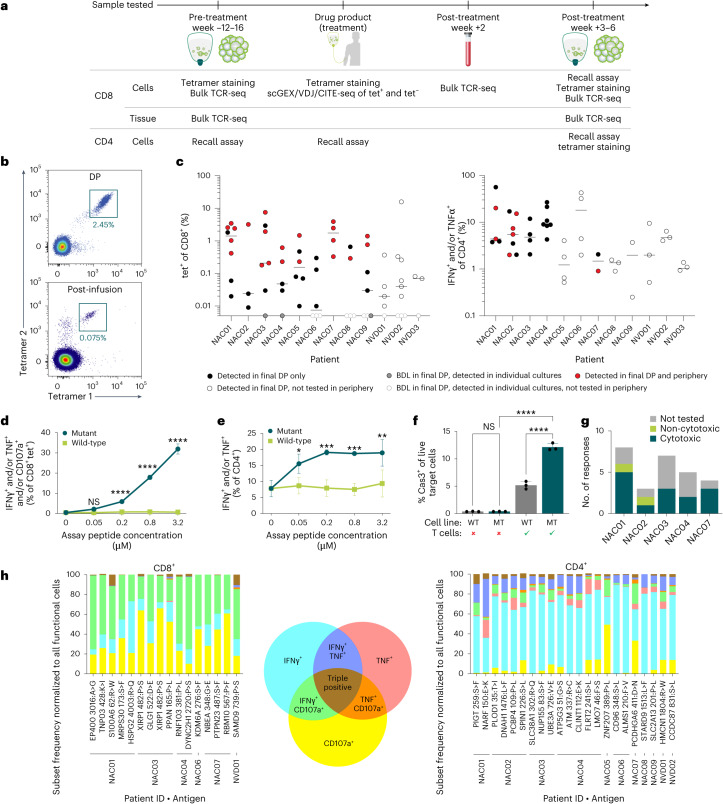
Polyclonal T cell responses in DP are mutant specific, polyfunctional and can be detected in the periphery. **a**, Overview of samples and methods used for characterization and tracking of CD4^+^/CD8^+^ responses. T cell responses and TCR clonotypes associated with the neoantigen-specific T cells are identified from the DP. Where sample was available, responses were tracked in patient tumors at week −16 and weeks +3–6 and in the peripheral blood at week −12, week +2 and weeks +3–6. The figure panel was created in BioRender (Gottstein, C. https://BioRender.com/k25z010 (2024)). **b**, Example flow cytometry plots of tet^+^ CD8^+^ T cells specific for mutant S100A6_62:R>W_ neoantigen in NAC01 in the DP (top) and at +3–6 weeks after infusion (bottom). Percentages are out of total CD8^+^ T cells. Fluorophores used in top panel were BV650 for tetramer 1 and BV421 for tetramer 2; fluorophores used in bottom panel were BV605 for tetramer 1 and PE for tetramer 2. **c**, Responses were detected in the DP using MHC class I tetramers for CD8^+^ responses and upregulation of IFNγ and/or TNF when rechallenged with mutant neoantigen peptide for CD4^+^ responses (*n* = 3, technical replicates). Responses shown as below detection limit (BDL) were detected in individual vessels before pooling but not in the final DP. After infusion, CD8^+^ T cells were detected using MHC class I tetramers, bulk TCR-seq and/or through rechallenge with neoantigen peptide to evaluate degranulation and/or secretion of TNF/IFNγ. Of the 12 patients with characterized DP, sufficient post-infusion PBMC sample was available from nine patients to track CD8^+^ responses by flow cytometry and bulk TCR-seq and from five to track CD4^+^ responses by flow cytometry. Most CD8^+^ responses (38/53) were tracked using both tetramer−based analysis and bulk TCR-seq in blood and bulk TCR-seq in tumor. The remaining responses (15/53) were tracked using tetramer staining in blood only. CD4^+^ T cells were detected using MHC class II tetramers and/or through rechallenge with neoantigen peptide to evaluate degranulation and/or secretion of TNF/IFNγ. To avoid the detection of false positives, tetramers that use different color combinations were used to confirm responses with an additional sample. Horizontal bar represents mean of all responses. A high frequency within the CD8^+^ responses correlated with detectability after infusion in periphery (red dots), as determined by unpaired two-sided *t*-test, *P* = 0.015. **d**, Representative data are shown for IFNγ and/or TNF-secreting and/or degranulating CD107a-positive CD8^+^ tet^+^ T cells in response to recall with different concentrations of mutant and wild-type PPAN_165:P>L_ from NAC03. Data are shown as mean with s.d. (*n* = 3, technical replicates) where *P* values are defined with a two-way ANOVA with Sidak’s multiple comparison test; *P* values: NS: *P* = 0.13; ****P ≤ 0.0001. **e**, Representative data are shown for IFNγ and/or TNF-secreting CD4^+^ T cells in response to recall with different concentrations of mutant and wild-type GANAB_658:E>G_ from NAC03. Data are shown as mean with s.d. (*n* = 3, technical replicates) where *P* values are defined with a two-way ANOVA with Sidak’s multiple comparison test; *P* values for increasing concentrations: NS: *P* > 0.99; *P = 0.03; ***P = 0.0004; ***P = 0.0003; **P = 0.002. **f**, A cytotoxicity assay was performed using A375 target cell lines (lentiviral transduced) expressing either the relevant wild-type or mutant neoantigen on the corresponding HLA allele and co-cultured with CD8^+^ T cells from the DP (bead isolated). Upregulation of caspase 3 (Cas3) on target cells was used to assess the killing capacity. Representative response for patient NAC03 to mutant and wild-type PPAN_165:P>L_ is shown. MT, mutant; WT, wild-type. Data are shown as mean with s.d. (*n* = 3, technical replicates) where *P* values are derived by transforming percentages into proportions, and an additive log ratio transformation was applied before performing a two-tailed Student’s *t*-test (NS: *P* > 0.99; ****P ≤ 0.0001). **g**, Summary of all DP CD8^+^ responses tested for killing capacity through the cytotoxicity assay. **h**, Summary of polyfunctionality data for all responses tested in the DPs. All graphs are normalized to total functional cells, which are defined as cells expressing/secreting any single or combination of IFNγ, TNF and/or CD107a. Left panel: CD8^+^ responses; right panel: CD4^+^ responses. Abbreviations for patient IDs: NAC, neoantigen cell dose received; NVD, never dosed. NS, not significant.

To learn more about the parameters that potentially contributed to success of this treatment approach, detailed translational analysis on all patients was performed (Fig. 3a). Because CD8^+^ and CD4^+^ T cells contribute to the proposed mechanism of action of BNT221 through direct cytotoxic and/or helper functions^40^, we set out to identify and enumerate CD8^+^ and CD4^+^ T cell responses in the DPs. CD8^+^ T cells were analyzed by flow cytometry using tetramers specific for each peptide in the manufacturing process predicted to bind to MHC class I. Neoantigen-specific CD4^+^ T cells were identified through an overnight co-culture assay, referred to as recall assay or recall response assay in this paper, in which the production of cytokines was measured to determine neoantigen reactivity in response to co-culture with peptide-loaded APCs.

The samples for these analyses were either from individual cell culture vessels in the final steps of manufacturing or from the final DP, and most analyses were carried out with DPs of all enrolled patients (*n* = 12). NEO-STIM generated multiple CD8^+^ and CD4^+^ T cell responses at various magnitudes in every DP (Fig. 3b,c and Supplementary Fig. 2). Also, most individual vessels contained at least one neoantigen-specific T cell response (58 of 65 vessels; Supplementary Data 1). Across 12 DPs manufactured, up to 13 total T cell responses were generated per DP, with a median of 5.5 CD8^+^ responses (range 3–9) and 3.5 CD4^+^ responses (range 2–7) (Fig. 3c and Supplementary Data 1).

#### Determination of de novo and memory responses in DP

The generation of de novo responses against patients’ neoantigens is a unique feature of NEO-STIM as it leads to a more diverse population of T cells that can potentially reduce the chance of antigen escape^41,42^. We, therefore, evaluated whether T cell responses generated through NEO-STIM were de novo (not detectable in the patient’s blood or tumor before treatment and presumably originating from the naive compartment) or pre-existing (detectable before treatment, stemming from the memory compartment).

Neoantigen-specific responses detected in the DPs were tracked in the blood (pre-infusion and post-infusion) using tetramer staining and recall assays in all treated patients (*n* = 9). When possible, CD8^+^ neoantigen-specific T cells were isolated from the DP, and single-cell GEX/VDJ/CITE sequencing (scGEX/VDJ/CITE-seq) was used to phenotypically characterize and identify TCR clones associated with the neoantigen responses. Bulk TCR sequencing (TCR-seq) was then performed on pre-infusion and post-infusion blood and tumor samples to track these TCR clones (Fig. 3a).

NEO-STIM generated multiple CD8^+^ T cell responses in every DP (ranging 3–9 per patient). Of 53 CD8^+^ responses detected, 22 were pre-existing (median 2, range 0–6 per patient). We, therefore, infer that 31 CD8^+^ responses (58.5%) were de novo (median 4, range 0–5 per patient) (Extended Data Table 2 and Supplementary Data 1). A total of 38 CD4^+^ responses were identified by recall response in the DPs (median 4, range 2–7 per DP), none of which were detected in the blood before infusion (Extended Data Table 2 and Supplementary Data 1). The number of pre-existing CD8^+^ and CD4^+^ T cell responses may be an underestimation due to their very low frequencies in the peripheral blood before infusion and the limit of detection of the assays used^21,43–45^. Taken together, these data demonstrate that NEO-STIM frequently raises T cell responses from the naive compartment and expands pre-existing T cell responses.

#### T cell specificity, cytotoxicity and polyfunctionality in DP

T cells show an impressive ability to respond to very few peptide–MHC molecules on the cell surface and discriminate between single amino acid substitutions, characteristic of the minimal protein changes found in neoantigens^46^. Assessment of the T cells’ ability to discriminate between the mutant and wild-type epitopes was performed to evaluate the potential for off-target effects. Reactivity and specificity toward the mutant form of the epitope was evaluated by co-culturing neoantigen-specific T cells overnight with autologous, peptide-loaded dendritic cells (DCs). The expression of cytokines and/or degranulation in response to either the mutant or wild-type peptides in a recall assay was measured (Fig. 3d,e and Supplementary Figs. 3 and 4). Although 100% of responses were shown to be mutant reactive (*n* = 44 CD4^+^ and CD8^+^ responses), eight responses displayed reactivity toward the wild-type peptide at high concentrations; however, wild-type reactivity was significantly lower compared to mutant peptide reactivity (Supplementary Fig. 4 and Supplementary Data 2).

Next, the potential of neoantigen-specific CD8^+^ T cells to kill target cells was evaluated by a flow cytometry–based cytotoxicity assay. T cells from five DPs, representing multiple responses, were incubated with antigen-expressing tumor cells in vitro. Upregulation of caspase 3 on the targets was measured as a readout for cytotoxicity. Fourteen of the 16 CD8^+^ responses (88%) were cytotoxic in response to target cells expressing the mutant neoantigen (Fig. 3f,g).

The ability of a T cell to co-express multiple functional molecules has been associated with better immune control in infectious disease, vaccines and cancer^47,48^. Polyfunctionality was, therefore, evaluated in a recall assay, based on production of interferon gamma (IFNγ) and/or tumor necrosis factor (TNF) and/or CD107a degranulation. This analysis revealed that all T cell responses exerted multiple effector mechanisms (Fig. 3h). At least two functionalities (typically IFNγ and CD107a) were detected in a large proportion of neoantigen-specific CD8^+^ T cells, and, although IFNγ secretion dominated in neoantigen-specific CD4^+^ T cells, most responses had a subset of T cells able to secrete both IFNγ and TNF. Collectively, these data show that NEO-STIM-induced T cell responses are highly specific, cytotoxic and polyfunctional.

#### Phenotypic and transcriptomic T cell analysis

The impact of T cell phenotype and its impact on expansion, persistence and anti-tumor response is an active field of study^49,50^.

**Extended Data Fig. 2:**
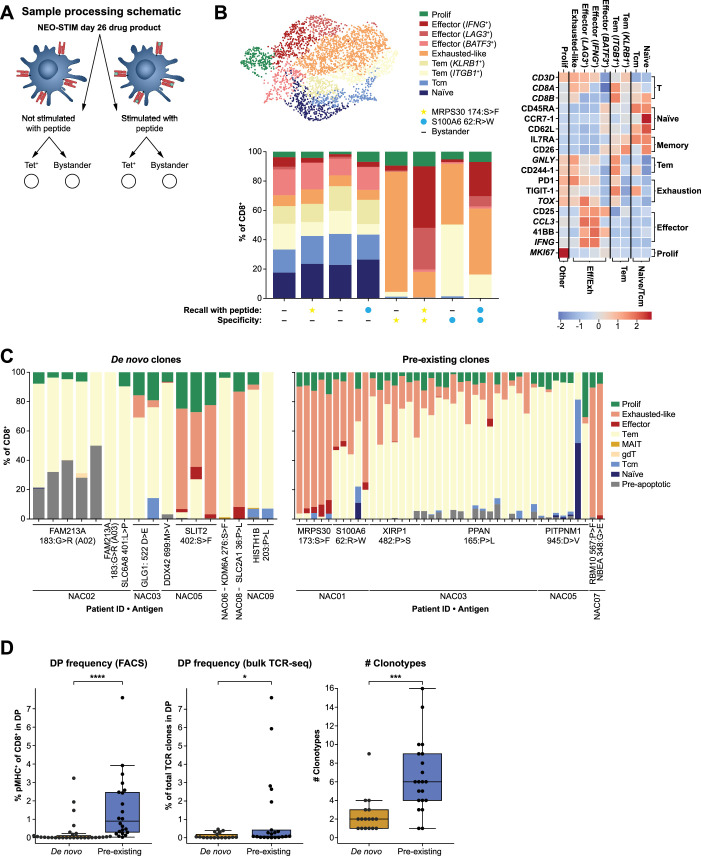
Neoantigen-specific T cells have diverse phenotypes. Extended Data Fig. 2: Neoantigen-specific T cells have diverse phenotypes. **a**. Sample treatment schematic for panel B. The figure panel was created in BioRender (Gottstein, C. https://BioRender.com/e86m166 (2024)). **b**. UMAP plot of multimodal single-cell data (GEX, CITE, VDJ) (top), cell type distribution (bottom), and key cell type markers (right) in drug product (DP) for representative patient NAC01. The grouping on the right *y* axis of the heatmap is based on current understanding of marker functions (left y axis). Exhausted-like cells were identified, among other features, by high PD-1 and low/absent IL-7RA expression. Italicized markers shown in the heatmap are RNA; non-italicized markers are CITE-seq surface proteins. See Supplementary Fig. 6 for further detail. **c**. Cell type distribution across de novo responses (not detected pre-treatment; left) or pre-existing responses (detected pre-treatment; right). **d**. DP frequency by FACS, bulk TCR-seq and number of clonotypes across responses from de novo (orange) or pre-existing (blue) responses. Boxplots show median and 25th and 75th data quartiles. Outliers (whiskers) were defined as 1.5 times the interquartile range. Number of responses across nine patients: *n*=29;22 (left) and *n*=17;21 (middle and right). Two-sided Mann-Whitney U test was used for statistical analysis; *P=0.02; ***P=0.0002; ****P=0.000004. Abbreviations for patient IDs: NAC: neoantigen cell dose received.

To understand T cell phenotypes at a transcriptional level, T cells were stimulated with or without relevant peptide and then sorted into tetramer positive and negative (tet^+^ and bystander) populations. Each subpopulation was subsequently processed for downstream multimodal scGEX/VDJ/CITE-seq (Extended Data Fig. 2a). After unsupervised clustering based on transcriptome and surface protein expression, we assigned phenotypic cell labels using standard markers (Extended Data Fig. 2b) and validated the assignments by comparing them to published signatures (Supplementary Fig. 5a–c)^49,51–53^. Effector T cells were characterized by both activation and dysfunction signatures, whereas exhausted-like T cells only displayed signatures of dysfunction^54–57^ (Supplementary Fig. 5a–c). These distinct transcriptional profiles resulted in the identification of unique cell subsets despite overlapping expression of common markers, such as PD-1.

Bystander cells showed a range of phenotypes, including naive, central memory, effector memory and effector-like, with a consistently high proportion of T central memory (Tcm) and naive, whereas unstimulated tet^+^ cells were largely effector memory or exhausted-like phenotypes. Stimulating tet^+^ cells with their cognate peptide resulted in an increase in effector phenotypes, in addition to preservation of some exhausted-like cells, indicating that phenotypes can shift upon antigen encounter (Extended Data Fig. 2b and Supplementary Fig. 6). Clonotypes within each response mostly showed a similar range of phenotypes, whereas phenotypes by response varied, even within the same patient (Extended Data Fig. 2c).

We showed above that most of the generated CD8^+^ T cell responses originated from the naive compartment (31/53 responses; Extended Data Table 2); however, when it comes to abundance in the DP, pre-existing responses were higher in frequency than de novo responses, with a higher number of clonotypes associated with each response (Extended Data Fig. 2d).

**Extended Data Fig. 3:**
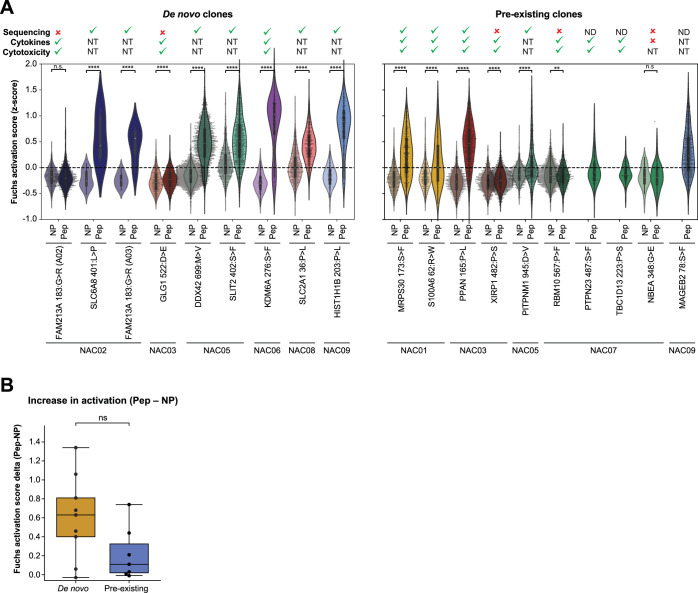
Neoantigen-specific T cells upregulate a transcriptional activation signature when stimulated with cognate antigen. **a**. Activation scores across all responses sequenced. Sequencing: Defined as a response with a significant difference in score between NP and Pep of ≥ 0.1. Statistical evaluation by two-sided t-test without multiple comparison adjustments. NS:*P*=0.37 (left) and *P*=0.96 (right); **P=0.005; ****P<0.00001. Fuchs activation score is a cell-level gene set score for T cell genes upregulated in response to peptide recall with viral peptides^54^. See Supplementary Fig. 5 for reference. Cytokines: Defined as T cells producing TNF, IFNγ, and/or CD107a in response to peptide in recall response assay, compared to NP control, as assessed in drug product (DP) characterization experiments. Cytotoxicity: Defined as T cells killing target cells expressing respective neoantigens in cytotoxicity assay of DP characterization experiments. Pre-existing responses and de novo responses determined by bulk TCR-seq and/or tetramer-based flow cytometry in the pre-infusion sample. Note that NAC03 de novo response showed statistical significance when comparing sequencing signatures of NP and Pep, but difference was less than 0.1. NT: Not tested. ND: No data (applies to sequencing responses with only one sample). NP – no peptide; Pep – stimulated with peptide. **b**. Statistical evaluation of difference in activation scores between all de novo and pre-existing responses. Boxplots show median and 25th and 75th data quartiles. Outliers (whiskers) were defined using 1.5 times the interquartile range. Two-sided Mann-Whitney U test was applied to calculate statistical significance. NS:*P*= 0.11. *n*=9;7 for de novo; pre-existing. NP – no peptide; Pep – stimulated with peptide. Abbreviations for patient IDs: NAC: neoantigen cell dose received.

Functional activity was determined by the increase in a transcriptomic activation signature^54^ upon antigen exposure (Extended Data Fig. 3). Increased functional activity was observed for most of both pre-existing (4/7) and de novo (7/9) responses that were evaluated (Extended Data Fig. 3a). There was a trend for higher activation delta for de novo responses versus pre-existing responses, with a median of 0.63 (s.d. 0.42) versus 0.11 (s.d. 0.26) (Extended Data Fig. 3b). The observation that exhausted-like tet^+^ T cells were able to respond to antigen indicates that neither de novo nor pre-existing responses are terminally exhausted.

#### Functional avidities of neoantigen-specific TCRs

**Extended Data Fig. 4:**
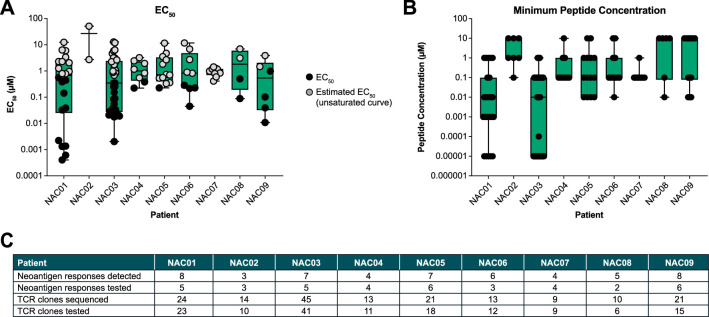
Neoantigen-specific TCRs show diverse functional avidity. **a**. Neoantigen-specific VDJ regions from TCRs of the DP were sequenced, alpha and beta chain RNA generated, and transfected into NFAT Jurkat cells to measure functional avidity (n=4, technical replicates). EC_50_: Peptide concentration resulting in half maximal effect. EC_50_ datapoints from saturated curves in black; estimated EC_50_ datapoints from unsaturated curves in gray. Boxplots show median and 25th and 75th data quartiles. Outliers (whiskers) were defined using 1.5 times the interquartile range. **b**. Minimum peptide concentration required for NFAT activation (*n*=4, technical replicates). Boxplots as in A. **c**. Row 1: Number of total CD8^+^ neoantigen-specific responses detected in the drug products across all patients; row 2: Number of CD8^+^ responses for which material was available for sequencing and avidity testing. Row 3: Number of TCR clones sequenced; note that there may be more than one TCR clone per neoantigen. Row 4: Number of TCR clones tested for functional avidity. The subset of TCR clones that were found to be neoantigen-specific is shown in panel A and B. TCR clones reported for EC_50_ values (A): *n*=22, 2, 40, 8, 12, 9, 9, 4, 6, and minimum peptide concentration (B): *n*=23, 7, 40, 9, 14, 11, 9, 6, 14 for NAC01-NAC09. Abbreviations for patient IDs: NAC: neoantigen cell dose received.

TCR avidity is known to be a key driver for T cell activity^58^. We evaluated functional avidity of neoantigen-specific TCRs isolated from the DP to understand the range of TCR avidities that are generated by NEO-STIM (Supplementary Data 3). RNA encoding each TCR was transfected into a NFAT-reporter Jurkat T cell line. TCR^+^ cells were co-cultured with APC lines loaded with peptide, and luciferase was quantified as a measure of T cell activation. Peptide concentrations for half-maximal effect (EC_50_) were determined for multiple clones from nine patients (median 9, range 2–40 per patient) (Extended Data Fig. 4a,c). We observed a broad range of functional avidities, from the nanomolar range to more than 10 µM (Extended Data Fig. 4a). This was true across multiple epitopes as well as within a single epitope. In some cases, the dose–response curve did not reach saturation; therefore, no true EC_50_ could be calculated, and estimated EC_50_s (gray dots in Extended Data Fig. 4a) are likely underestimated. Therefore, we also plotted the minimum peptide concentration required for T cell activation (Extended Data Fig. 4b). These data show that NEO-STIM generates a polyclonal T cell product with a range of functional avidities.

#### Pharmacodynamics of neoantigen-specific T cells

Long-term persistence has been associated with good clinical outcome in ACT^49,59^. To evaluate persistence of the T cell responses identified in BNT221, patients’ PBMCs and tumor samples were tested 2–6 weeks after infusion by flow cytometry and/or bulk TCR-seq, as described in Fig. 3a. All but one treated patient had a measurable T cell response after infusion from those detected in the DP (Fig. 3b,c). Across all treated patients, 48% of CD8^+^ responses and 28% of CD4^+^ responses detected in the DP were also detected in the peripheral blood after infusion (CD8^+^: 21/44 tested, median 2; CD4^+^: 7/25 tested, median 1) (Fig. 3c). Additionally, 43% of CD8^+^ responses were detected in the tumor after infusion (CD8^+^: 13/30 tested, median 2; CD4^+^: not tested in tumor after infusion) (Extended Data Table 2). Notably, the CD8^+^ T cell responses that had the highest frequency in the DPs were also the ones that were detected in the periphery after infusion (Fig. 3c).

**Fig. 4:**
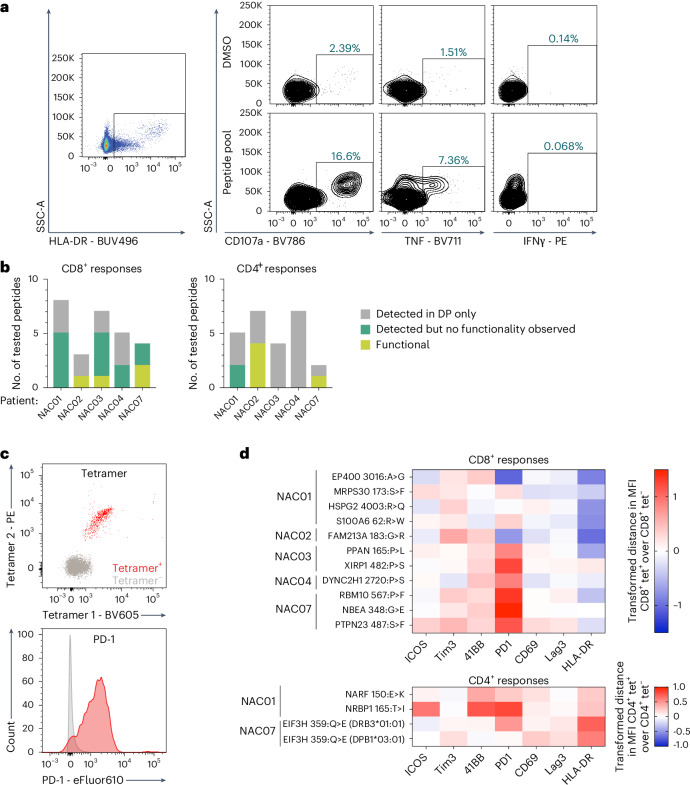
A subset of post-infusion responses are functional. **a**, Example flow cytometry plots to illustrate the experimental approach. NAC02 PBMCs were recalled with no peptides (DMSO) or with a peptide pool consisting of all short and long epitope peptides for which responses were detected in the DP. Cells were then stained for HLA-DR, IFNγ, TNF and CD107a for assessing functionality. Left, for CD4^+^ T cells, gating on cells with high HLA-DR expression enriched for cells considered reactive to the recalled peptide(s) and, therefore, neoantigen specific; right, HLA-DR^+^ CD4^+^ cells were further analyzed for IFNγ, TNF and/or CD107a expression. **b**, Summary of functionality profiles for all patients where functionality of T cell responses (CD8^+^ and CD4^+^) was tested in the periphery at +3−6 weeks after infusion. Responses are considered functional if expression of IFNγ, TNF or CD107a is at least 1.5-fold greater in the peptide recall condition compared to no peptide recall (DMSO). **c**, Post-infusion (weeks +3–6) PBMCs were stained with tetramers and surface antibodies for flow cytometry analysis. No stimulation or recall was performed. Example flow plot, with upper panel showing tet^+^ cells specific for XIRP1_482:P>S_ in red and tet^−^ cells in gray. CD8^+^ cells were gated according to tetramer positivity and surface expression of activation/exhaustion markers. Lower panel shows mean fluorescence intensity of PD-1 surface expression of XIRP1_482:P>S_^+^ (red, CD8^+^ tet^+^) and XIRP1_482:P>S_^−^ (gray, CD8^+^ tet^−^) T cells from NAC03. **d**, The heatmap shows transformed distance in MFI of tet^+^ over tet^−^ CD8^+^ T cells (top) and CD4^+^ cells (bottom). Positive values (red) indicate upregulation, and negative values (blue) indicate downregulation. *x* axis: surface markers that were quantified in the flow cytometry panel; *y* axis: patient IDs and the corresponding neoantigen epitopes. For NAC07, the EIFH3_359:Q>E_ epitope was presented on two different class II alleles, and both are shown in the heatmap. Abbreviations for patient IDs: NAC, neoantigen cell dose received; NVD, never dosed. SSC-A, side scatter area.

Functionality of responses detected at 3–6 weeks after infusion was evaluated in a recall assay, based on production of IFNγ and/or TNF and/or CD107a degranulation. CD4^+^ cells were first gated on HLA-DR to identify activated T cells, followed by gating on functional markers (Fig. 4a). Functional neoantigen-specific T cell responses were detected in post-infusion PBMCs (median 1, range 0–5) for all NAC01 (five patients tested: NAC01–NAC04 and NAC07) (Fig. 4b). Across these five patients, a total of 24 CD8^+^ and CD4^+^ responses were detected in post-infusion samples, and, of these, nine were functional (38%, 4/17 CD8^+^ and 5/7 CD4^+^ responses) (Fig. 4b).

A phenotypic flow panel used to analyze neoantigen-specific T cell responses directly ex vivo revealed that PD-1 was upregulated after infusion on the majority of tet^+^ CD8^+^ and tet^+^ CD4^+^ T cells compared to tet^−^ cells (Fig. 4c,d; five patients, 11 CD8^+^ and 4 CD4^+^ responses tested). These tet^+^ CD4^+^ and CD8^+^ T cells were primarily central and/or effector memory phenotype (Supplementary Fig. 7). Our data suggest that a subset of responses detected in the DP persist after infusion, remain functional and express PD-1.

#### Immune analysis of NAC09 with tumor regression after BNT221

**Fig. 5:**
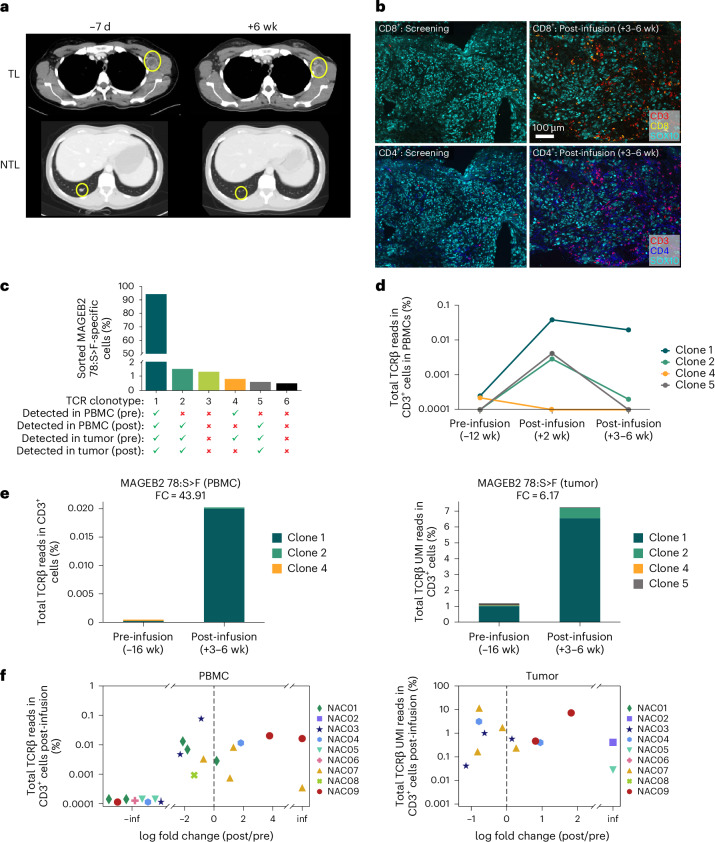
Case study: patient NAC09—tumor reduction, T cell infiltration and neoantigen-specific T cells from DP in periphery and tumor. **a**, CT scans from NAC09. Circled regions highlight the target (top) and non-target lesions (bottom) with observed regression between day −7 and week +6. NTL, non-target lesion; TL, target lesion. **b**, Multiplexed immunofluorescent images of tumor pre-infusion and post-infusion for NAC09 showing CD3^+^, CD8^+^ and SOX10^+^ (top) and CD3^+^, CD4^+^ and SOX10^+^ (bottom) cells. SOX10, a melanoma marker, highlights the tumor cells. Representative images from a total of 20 regions of interest per tissue are shown. **c**, Detection of MAGEB2_78:S>F_-specific clonotypes in the periphery and in the tumor using bulk TCR-seq. Clonotypes were identified from NAC09 DP using tetramer sorting, followed by scGEX/VDJ/CITE-seq. Bar plot shows frequencies of each clonotype in the DP. **d**, Percentage of MAGEB2_78:S>F_-specific clonotypes from DP in peripheral blood using bulk TCR-seq of the TCR β-chain. Clonotypes with percentages below the limit of detection for the assay are shown at 0.0001%. **e**, Percentage of MAGEB2_78:S>F_-specific clonotypes of total TCRs detected in the peripheral blood (left) and tumor (right) pre-infusion and post-infusion using bulk TCR-seq. Note that clone 5 was detected at week +2 but not at weeks +3–6. **f**, log fold change (FC) of T cell responses for all responses tested in the blood pre-infusion and post-infusion (weeks +3–6) by bulk TCR-seq. inf, infinite fold change (positive: responses that were detected post-treatment only; negative: responses that were detected pre-treatment only). Left, peripheral blood; right, tumor. Clonotypes with percentages below the limit of detection for the assay are shown at 0.0001%. Abbreviations for patient IDs: NAC, neoantigen cell dose received. wk, weeks.

The tumor reductions were observed directly after BNT221 administration in all cases where they occurred, suggesting a causative relationship (Fig. 2b). However, when we evaluated correlations of potential factors that could impact clinical outcome (such as dose, fraction of antigen-specific T cells, number of epitopes recognized and functionality), we did not observe clear correlations comparing these factors in patients with tumor reduction to those without. We then decided to perform a detailed assessment for patient NAC09, given that this patient had the largest tumor reduction of patients evaluated in this study. Six-week post-infusion computed tomography (CT) evaluations for this patient revealed a 20% reduction of the target lesion in the left axilla as well as a significant reduction in the non-target lesions, which included a secondary axillary lymph node and millimetric pulmonary lesions (Fig. 5a). Concurrent immunohistochemistry analysis for this patient demonstrated an increase in T cell infiltration in the tumor. Specifically, CD4^+^ and CD8^+^ T cell density increased 14-fold and 12-fold, respectively, compared to the screening tumor biopsy (Fig. 5b).

Bulk TCR-seq data from blood and tumor were subsequently analyzed to quantify TCR clonotypes from the DP throughout the course of treatment. For each response, clonotypes were tracked in both the blood and the tumor. Within the MAGEB2_78:S>F_-specific T cell response—one of the six responses that could be tracked in this patient—six clonotypes were identified in the DP. Analysis in tumor demonstrated that four of these were detected pre-infusion, and, among these, three were detected post-infusion (Fig. 5c). These three clonotypes increased approximately sixfold in the tumor and approximately 44-fold in the periphery from pre-infusion to post-infusion. (Fig. 5d,e). NAC09 was the only patient with multiple responses (MAGEB2_78:S>F_ and ATXN2_1261:T>A_) showing post-infusion expansion in both the blood and the tumor (Fig. 5f).

**Extended Data Fig. 5:**
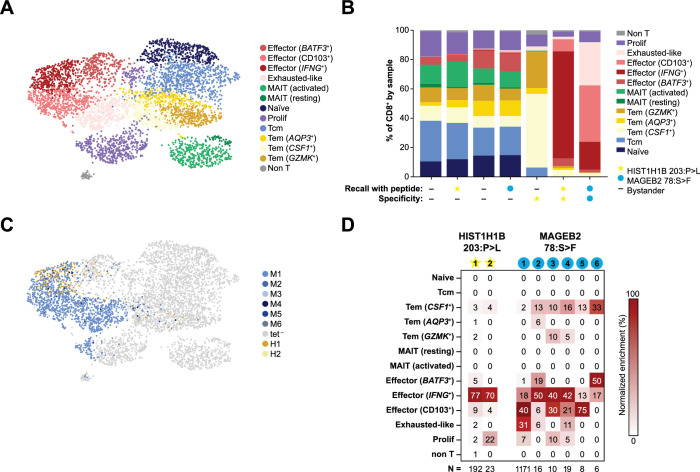
Transcriptional differences between MAGE-B278:S > F and HIST1H1B203:P > L responses. **a**. UMAP of multimodal single-cell data from Patient NAC09 drug product (GEX, CITE, VDJ). **b**. Percentages of single-cell phenotypes out of all CD8^+^ T cells for each experimental condition from NAC09 drug product. **c**. UMAP highlighting MAGEB2_78:__S__>__F_-specific (blue) and HIST1H1B_203:__P__>__L_-specific (yellow) clonotypes. All highlighted cells underwent antigen recall with peptide prior to sequencing. Tet^−^: bystander cells. **d**. Heatmap showing phenotypes of HIST1H1B_203:__P__>__L_-specific cells (left) and MAGEB2_78:__S__>__F_-specific cells (right) after antigen recall by clonotype. Abbreviations for patient IDs: NAC: neoantigen cell dose received.

A T cell response toward HIST1H1B_203:P>L_ was also detected in the DP from patient NAC09 but did not expand post-infusion. To better understand these functional differences, we evaluated transcriptional signatures of the six MAGEB2_78:S>F_ and two HIST1H1B_203:P>L_ clonotypes. Most clonotypes displayed a mix of effector−like (primarily *IFNG*^+^) and exhausted-like phenotypes when stimulated with peptide (Extended Data Fig. 5a–d), irrespective of whether they came from the expanded or the non-expanded response.

These data suggest that post-treatment expansion of neoantigen-specific T cells may be a contributor to the tumor regression observed in this patient. Additional data in future patients will be valuable to further validate this observation.

## Discussion

ACT is an emerging treatment modality for solid tumors, notably including recent major advances in TCR-T applications and clinical approval for TIL therapy^16,27^. Although NEO-STIM shares many characteristics with these approaches, our data suggest conceptual advantages of our therapy. We developed a product based on expansion of neoantigen-specific T cells that was well tolerated and showed signs of clinical activity, with SD as best overall response. The infused T cells were highly specific, polyfunctional and cytotoxic and expressed TCRs with a wide range of avidities. Collectively, these findings are a critical step toward establishing an additional treatment paradigm for patients with advanced melanoma.

Neoantigen-specific T cell reactivity detected in patients is often limited to a few epitopes, even though the mutational landscape of each patient’s tumor is broad^43,60–63^. NEO-STIM reproducibly generated DPs with multiple neoantigen-specific CD8^+^ and CD4^+^ T cell responses, providing enhanced diversity over the restricted, native immune response. In addition to inducing de novo T cell responses, our data show expansion of pre-existing T cell responses, validating the neoantigens selected as both physiologically relevant and immunogenic.

Key measures of T cell quality are their functionality and phenotype. Although most T cell responses were a mixture of memory and exhausted-like phenotypes, they did show an activation signature upon antigen engagement. These observations confirm that neoantigen-specific T cells from the circulation are not terminally exhausted. This is consistent with previous findings where functionality of T cells from tumors and peripheral blood was directly compared and where T cells from the periphery generally showed a trend toward higher functionality^19^.

TCR avidity is widely recognized as a major attribute promoting an anti-tumor immune response^47^. Moreover, it has been shown that TCR signal strength is a driver of clonal behavior, forcing the development of T cells with distinct phenotypic and functional traits^64^. It is hypothesized that an optimal anti-tumor T cell response is polyclonal, balancing persistence, effector and memory functions. We showed that TCRs isolated from BNT221 had a broad range of functional avidities toward multiple neoepitopes, and this diversity was also true among TCRs specific for the same neoantigen. Although avidities of TIL-derived TCRs are generally higher^65^, the polyclonal profile of BNT221 is a key differentiator compared to TCR-T therapies and forms a solid starting point for continued development.

BNT221 treatment was well tolerated. The vast majority of AEs observed were attributed to the lymphodepletion chemotherapy preceding BNT221 infusion. If confirmed in larger trials, this safety profile will be a major advantage relative to other ACTs. Although the BNT221 activity signals in this phase 1 clinical trial were less pronounced than responses seen with established ACTs, the tumor regressions observed in several patients are nevertheless of interest, considering this heavily pre-treated metastatic melanoma population.

The relatively small number of treated patients and the lack of large effect sizes for tumor responses make correlating DP characteristics to clinical outcomes difficult. Additionally, comparisons between patients are complex due to the personalized nature of the product, which includes variances in infused cell dose, fraction of neoantigen-specific responses and characteristics of targeted mutations.

From a clinical perspective, the total time to manufacture the cell product is still a limitation, although it is already improved compared to previously reported similar approaches^27^. The need to improve product expansion, persistence and potency and to reduce the total time until infusion are recognized as key objectives for future clinical development. In particular, experience with TIL and chimeric antigen receptor (CAR)-T cell therapy has shown that post-infusion expansion is critical for clinical efficacy^66–69^. In our study, robust expansion of multiple neoantigen-specific T cell responses in both tumor and peripheral blood 2–6 weeks after infusion was limited to the patient with the most pronounced tumor regression (NAC09). One means to accomplish such improvements is through expression of constitutively active cytokine receptors to mediate improved T cell survival and expansion during in vitro manufacturing and in vivo post-infusion^70^. Other approaches include overexpression of genomic targets that increase persistence and limit exhaustion, such as *FOXO1* or c-Jun (*JUN*), or deleting genes such as *PTPN2*, which is associated with T cell exhaustion^71–73^. The introduction of switch receptors to convert an inhibitory pathway into a T cell co-stimulatory pathway—for example, PD1-41BB—is also of interest^74^.

Combination strategies are another approach to enhance potency, expansion and persistence. Combining BNT221 with a cancer vaccine may mediate controlled expansion of the tumor-specific T cells, as demonstrated in a CARVac approach^75^. Our observation of PD-1 upregulation on multiple post-infusion neoantigen-specific T cell responses provides a strong rationale for PD-1 inhibition, and this combination is currently in clinical testing.

In summary, we demonstrated that the generation of this robust neoantigen cell therapy is feasible and generates a diverse, highly functional product. Future studies with improved product iterations and combination approaches hold great promise for this new cell therapeutic approach across solid tumors with high mutational burden.

## Methods

### Clinical study design and execution

#### Inclusion and ethics

This study was approved on 24 April 2020 by the Central Committee on Research Involving Human Subjects (approval number NL72301.000.19). It is executed at the NKI in accordance with the Declaration of Helsinki and International Council for Harmonization Good Clinical Practice (ICH GCP) guidelines. Informed consent was obtained from all patients.

#### Study design

NCT04625205 is an open-label FIH phase 1 clinical study to investigate the safety and efficacy of BNT221 in participants with locally advanced or metastatic melanoma. All participants must have either progressed after ICB treatment or not be eligible for ICB. Participants eligible for BRAF-targeted treatment must have had prior treatment with BRAF and/or MEK inhibitors.

This study is conducted in two parts:

Part 1

Part 1 is a dose-finding study for BNT221 with a monotherapy arm (3 + 3 study design, two DLs, minimum of three patients per DL) and combination treatment arms, evaluating BNT221 co-administered with a PD-1 inhibitor or interleukin (IL)-2. Monotherapy DLs tested were 1 × 10^8^–1 × 10^9^ total cells (DL1) and 2 × 10^9^–1 × 10^10^ total cells (DL2). Three patients were assigned to DL1 and six to DL2. Previous studies with TIL-based therapies were considered in devising a starting dose and dose range for this study. Out of safety considerations, DL1 of the cell product for BNT221 was chosen at substantially lower doses than demonstrated to be tolerable in TIL studies^76^. As an additional safety precaution, within dose cohorts, enrollment of the first three patients was staggered at a minimum of 2-week intervals. The highest planned dose was chosen taking manufacturing feasibility into account, facilitating consistent and reproducible manufacturing.

The part 1 monotherapy arm is completed, from which we report a non-pre-specified analysis here.

The part 1 combination therapy arm with a PD-1 inhibitor is currently ongoing.

Part 2

Part 2 is an expansion cohort of BNT221 administered with a PD-1 inhibitor with or without CTLA-4 inhibitor in the setting of first-line metastatic disease.

Primary objectives for part 1 were evaluation of safety and identification of highest tolerable dose. Endpoints were AEs, SAEs and AEs leading to treatment discontinuation as well as changes in laboratory values, physical examination findings and vital signs.

The secondary objective of the study is evaluation of anti-tumor activity, and endpoints include overall response rate based on RECIST 1.1, DOR, CBR and PFS.

Exploratory objectives included the characterization of the immune response before, during and after treatment and the characterization of the clonal expansion, persistence and phenotype of the transferred cells.

Inclusion criteria:

1. Adult (age 18–75 years) men and women willing and able to give written informed consent
2. Histologically confirmed unresectable or metastatic melanoma
3. Part 1:
  - Have previously received a PD-1/PD-L1 inhibitor (either as single agent or in combination) and a CTLA-4 inhibitor–containing regimen (single agent or combination) before BNT221 with disease progression after these therapies or otherwise lack of clinical benefit as determined by the study investigator.
    Note: Patients who have received a PD-1/PD-L1 inhibitor and ipilimumab (CTLA-4 inhibitor) are eligible; patients who have discontinued a PD-1/PD-L1 or a CTLA-4 inhibitor due to toxicity and those who are deemed not appropriate to receive a CTLA-4 inhibitor are eligible (except for part 1 cohort patients to receive additional PD-1 inhibitor therapy).
4. Part 2:
  - Have received/are currently receiving a PD-1/PD-L1 inhibitor (as a single agent or in combination with CTLA-4 inhibitor) for at least 3 months. Have documented SD by RECIST 1.1 or clinically asymptomatic PD on the most recent imaging assessment, which must have occurred within 3 months of enrollment. In the opinion of the investigator, are medically eligible and able to continue with PD-1/PD-L1 inhibitor therapy.
  - In the opinion of the investigator, would benefit from the addition of a T cell–based therapy.
5. For known BRAF-mutant patients: patients must have also received targeted therapy (BRAF inhibitor or BRAF/MEK combination therapy) before BNT221, unless deemed not appropriate to receive these treatments by the investigator.
6. Have at least one site of measurable disease by RECIST 1.1
7. At least one site of disease must be accessible to biopsy for tumor tissue for sequence and immunological analysis. The biopsy site may be the same as the measurable site so long as it remains measurable. Surgical resection of the measurable site may not be performed if that site is the only measurable lesion. An archival biopsy may be used in place if the biopsy was taken within 6 months of informed consent.
8. Have Eastern Cooperative Oncology Group Performance Status (ECOG PS) of 0 or 1
9. Recovered from all toxicities associated with prior treatment to acceptable baseline status (for laboratory toxicities, see below limits for inclusion) or a National Cancer Institute Common Terminology Criteria for Adverse Events (NCI CTCAE) version 5.0 grade of 0 or 1, except for toxicities not considered by the treating physician to be a safety risk (for example, alopecia).
10. Screening laboratory values must meet the following criteria and should be obtained before any production phase assessments:
  - White blood cell count ≥3 × 10^3^ per microliter
  - Absolute neutrophil count (ANC) ≥ 1.5 × 10^3^ per microliter
  - Platelet count ≥ 100 × 10^3^ per microliter
  - Hemoglobin > 9 g dl^−1^ or 6 mmol L^−1^
  - Serum creatinine ≤ 1.5× upper limit of normal (ULN) or creatinine clearance ≥ 50 ml min^−1^ by Cockcroft–Gault
  - Aspartate aminotransferase (AST) and alanine aminotransferase (ALT) ≤ 3× ULN
  - Total bilirubin ≤ 1.5× ULN (except in patients with Gilbert syndrome, who can have total bilirubin < 3.0 mg dl^−1^)
  - International normalized ratio, prothrombin time (PT) or activated partial thromboplastin time (aPTT) ≤ 1.5× ULN unless the patient is receiving anticoagulant therapy, as long as PT or aPTT is within therapeutic range of intended use of anticoagulants

Exclusion criteria:

1. Age older than 75 years or younger than 18 years
2. Received more than three prior lines of therapy for metastatic disease
3. Have an active or history of autoimmune disease (known or suspected). Exceptions are permitted for vitiligo, type 1 diabetes mellitus, residual hypothyroidism due to autoimmune condition requiring only hormone replacement, psoriasis not requiring systemic treatment or conditions not expected to recur in the absence of an external trigger.
4. Have known active central nervous system metastases and/or carcinomatous meningitis. Patients with previously treated brain metastases may participate provided they are stable (without evidence of progression by imaging (using the identical imaging modality for each assessment, either magnetic resonance imaging or CT scan) for at least 4 weeks before enrollment and any neurologic symptoms have returned to baseline), have no evidence of new or enlarging brain metastases and are not steroid dependent for at least 7 d before enrollment. This exception does not include carcinomatous meningitis, which is excluded regardless of clinical and/or radiographic stability.
5. Active systemic infections requiring intravenous antimicrobial therapy; coagulation disorders or other active major medical illnesses of the cardiovascular, respiratory or immune system, as evidenced by a positive stress thallium or similar test; myocardial infarction; clinically significant cardiac arrhythmias, such as uncontrolled atrial fibrillation, ventricular tachycardia or second-degree or third-degree heart block; and obstructive or restrictive pulmonary disease.
6. Active major medical illnesses of the immune system, including conditions requiring systemic treatment with either corticosteroids (>10-mg daily prednisone equivalents) or other immunosuppressive medications within 14 d before BNT221 infusion. Inhaled or topical steroids and adrenal replacement doses (≤10-mg daily prednisone equivalents) are permitted in the absence of active autoimmune disease.
7. Known HIV infection, active chronic hepatitis B or C and/or life-threatening illnesses unrelated to cancer that could, in the investigator’s opinion, interfere with participation in this study
8. Have any underlying medical condition, psychiatric condition or social situation that, in the investigator’s opinion, would interfere with participation in the study
9. Have a planned major surgery that is expected to interfere with study participation or confound the ability to analyze study data
10. Are pregnant or breastfeeding or expecting to conceive or father children within the projected duration of the study, starting with the screening visit through 150 d after the end-of-trial (EOT) visit. Nursing women are excluded from this study because there is an unknown but potential risk of AEs in nursing infants secondary to treatment of the mother with treatments to be administered in this study.
11. Have a history of another invasive malignancy aside from melanoma, except for the following circumstances:
  - Patient has been disease free for at least 2 years and is deemed by the investigator to be at low risk for recurrence of that malignancy
  - Patient was not treated with systemic chemotherapy for carcinoma in situ of the breast, oral cavity or cervix or basal cell or squamous cell carcinoma of the skin.

The high-level study design and treatment schedule are illustrated in Fig. 1a,d.

#### Treatment regimen

Before BNT221 treatment, pre-screening, screening, production and pre-infusion were performed. Eligibility was assessed during the pre-screening phase, and minimum tumor mutational burden was assessed during the screening phase.

PBMCs were obtained via leukapheresis and used for BNT221 production (see the ‘Generation of BNT221’ subsection). At the time of the BNT221 product release and after re-confirmation of eligibility, patients initiated a 5-d lymphodepletion regimen of cyclophosphamide, 25 mg kg^−1^ d^−1^ for 2 d, followed by fludarabine, 25 mg m^−^^2^ d^−1^ for 3 d. At the conclusion of the lymphodepletion regimen, BNT221 was administered by intravenous infusion (day +1) in an inpatient setting. Signs of toxicity were monitored in an in-hospital setting for up to 2 weeks after BNT221 infusion. After BNT221 treatment, filgrastim was administered subcutaneously and was continued daily until the neutrophil count levels were more than 1.0 × 10^9^ per liter for 3 d or more than 5.0 × 10^9^ per liter. When the neutrophil count reached this threshold level, the patient was discharged from the hospital.

In some instances, patients could, per the decision of the study investigator, receive melanoma-directed therapy before the BNT221 pre-infusion phase, but treatment was discontinued before BNT221 treatment (see Extended Data Table 2 for details).

#### Follow-up

After hospital discharge, patients were seen in clinic weekly for 4 weeks and then starting at week +6 every 6 weeks until week +36. They were also seen at week +52 for an EOT visit if the patient completed the study. Safety assessments included AE and SAE collection, symptom-directed physical examinations, measurement of vital signs, ECOG PS and safety laboratory assessments.

All imaging evaluations (for example, chest, abdomen, pelvis and brain) were completed per RECIST 1.1 guidelines. Per protocol, evaluations were scheduled at pre-screening, during production (week −12), at pre-BNT221 infusion day −7 and at post-infusion weeks +6, +12, +18, +24 and +36.

Tumor and blood samples were collected per trial schematic in Fig. 1d. Blood was collected at week +2, week +6 and then every 6 weeks in the absence of disease progression until week +36 post-infusion. Where possible, a leukapheresis and/or a tumor biopsy were collected between weeks +3 and +6 post-infusion for in-depth immunological studies as well as a tumor biopsy and blood draw at the end of treatment. Tumor samples were from the primary tumor (skin) or metastatic sites (liver, spleen and lymph nodes) for the initial screening sample. Tumor biopsies were taken at weeks +3–6 from five patients (NAC02, NAC03, NAC05, NAC08 and NAC09), and all but one (NAC08) were from the same site, or adjacent to it, as at initial screening. Four patients (NAC02, NAC04, NAC07 and NAC08) had a resection or biopsy at end of therapy, either from a subcutaneous site or from metastatic lymph nodes. Patient samples were processed and stored as described previously^37^.

### Generation of BNT221

#### WES

WES was carried out in a Clinical Laboratory Improvement Amendments/College of American Pathologists (CLIA/CAP)-accredited laboratory (Personalis). In brief, libraries were generated using the laboratory’s WES library protocol, ACE Cancer Research Exome, and its RNA-seq library protocol, ACE Cancer Research Transcriptome. Libraries were sequenced using an Illumina HiSeq. WES was conducted on a tumor sample and a normal blood sample per patient (depths: 280–417 reads and 141–165 reads per base pair, respectively). RNA-seq was conducted on tumor samples only (targeting 100 million paired-end reads, resulting in 12–316 million paired-end reads per library).

The HLA-A, -B, -C, -DR, -DP and -DQ genotype of each patient was determined from blood aliquots through conducting targeted amplification and Sanger-based sequencing (Versiti).

#### Alignment and mutation calling

All alignment, mutation calling and filtering and gene expression quantification were done in accordance with methods described previously^37^. In brief, the neoepitope selection goes through several selection steps: neoepitopes were first prioritized based on the expression of mutated alleles by RNA-seq, followed by binding to autologous HLA-A, HLA-B and HLA-C and additional variables. From this, the 40 top-scoring MHC class I neoepitopes (8–12 aa in length) and the 20 top-scoring MHC class II neoepitopes (25 aa in length) were then selected for manufacturing as described in the next subsection (Fig. 1b,c).

#### Peptide selection

Neoantigen-representing peptides were selected chiefly based on presentation likelihood scores that account for HLA binding potential, mutation expression and proteasomal processing potential. The corresponding prediction models were trained using mass-spectrometry-derived HLA ligandomics data^35,36^.

Beyond presentation score, several additional factors were considered. Specifically, we favored mutations with high cancer cell fraction (CCF) over those with low CCF; we favored frameshifts over point mutations (due to potential for greater immunogenicity); and we favored mutations in oncogenes (due to likely essentiality). These additional factors were implemented as scaling factors on the presentation scores and were constrained to a narrow range (±5%) so that presentation score would still be the dominant factor in epitope ranking. Hard rules were applied to exclude any mutations lacking RNA evidence and any mutations with a very low CCF (<10%).

Forty peptides were selected in total for MHC class I. MHC class I–targeting peptides were restricted to lengths 8–12 aa. A cap was imposed such that no single mutation could contribute more than three peptides to the peptide set. A downward scaling factor was applied to peptides predicted to bind HLA-C, such that HLA-C peptides would comprise only approximately 10% of peptide selections on average.

Twenty peptides were selected in total for MHC class II. MHC class II–targeting peptides were restricted to length 25 aa, with the mutations located within 4 aa of the peptide center. Consistent with MHC class I, no single mutation was permitted to account for more than three selected peptides. Also similar to MHC class I, we applied locus-level weights; the weights were tuned to select HLA-DR, -DP and -DQ peptides at an approximate 5:2:1 ratio. Finally, we enforced a constraint requiring the 20 selected peptides to include the five top-expressed mutations.

#### Good Manufacturing Practice peptide manufacturing

Good Manufacturing Practice (GMP)-grade peptides for BNT221 production were synthesized using solid-phase peptide synthesis (SPPS) on automated peptide synthesizers (PPLs). 9−fluorenylmethyloxycarbonyl (Fmoc) chemistry was employed. After the assembly of the peptide chains, the peptides were removed from the resins using cleavage cocktails containing trifluoroacetic acid and scavengers. The peptides were precipitated and washed with ether. After drying, the crude peptides were purified on semi-preparative high-performance liquid chromatography (HPLC) systems with ultraviolet (UV) detector. The HPLC fractions were analyzed using ultra-performance liquid chromatography (UPLC)-UV/MS systems to determine molecular weights (MWs) and purities. The fractions containing the target peptides and desired purities were lyophilized to solid powders with the final purities of at least 90%. Up to 40 short peptides (8–12 aa) and 20 long peptides (25 aa) per patient were formulated in DMSO and mixed into up to six pools, each containing up to 10 peptides. The pooled peptide solutions were sterile filtered through 0.2-μm filters and analyzed for visual appearance, identity, MWs (based on UPLC-UV/MS analysis), endotoxin, bioburden and strength before their releases from the GMP manufacturing site. The amino-termini and carboxyl-termini of the peptides are free amines and carboxylic acids, respectively, without structural modifications.

#### Patient leukapheresis collection and processing for manufacturing of DPs

PBMCs for production were collected via leukapheresis approximately 12 weeks before infusion and processed at Sanquin. Within 36 h of leukapheresis collection, the blood cells were washed with a saline-based wash buffer and cryopreserved in saline-based cryopreservation buffer containing DMSO in 2–4 bags for manufacturing and additional vials for material release and exploratory studies. All PBMC bags and vials were cryopreserved in a controlled rate freezer. PBMC bags were transferred to NKI-Antoni van Leeuwenhoekziekenhuis (NKI-AVL) for cell therapy manufacturing upon completion of GMP peptide starting material manufacturing.

#### Cell therapy manufacturing

Cell therapy manufacturing was performed at GMP facilities of the BioTherapeutics Unit at NKI-AVL. Cryopreserved patient PBMCs were thawed, washed, depleted of CD14^+^ and CD25^+^ subsets via magnetic separation, washed again and seeded in culture vessels with cell culture media comprising FLT3L (CellGenix) on day 0. Up to six parallel culture vessels were used per patient. Peptide pools with unique, personalized neoantigens were added to each culture vessel on day +1 of the manufacturing process and incubated for 1 h, and cultures were matured with media containing maturation cytokines TNF, IL-1β and IL-7 (CellGenix) and PGE-1 (Pfizer). Cultures were maintained with media exchange and cytokine additions (IL-7 and IL-15 (CellGenix)) through day +14 of the manufacturing process. On day +12 of the manufacturing process, a second set of patient PBMCs was thawed, and the process described above was repeated. On day +14, the cultures from day 0 were harvested, and cells were restimulated by combining with the matching cultures started on day +12, which used the same peptide pools. On day +26, each of the six restimulated cultures (containing all material started on day 0 and day +12) were combined, washed and formulated in a saline-based cryopreservation buffer containing DMSO. The formulated cells were added to a patient infusion bag and to vials for release and characterization testing. The patient infusion bag and vials were frozen in a controlled rate freezer and stored in liquid nitrogen until use.

#### Peptide synthesis for immunological assays

The peptides for in vitro immune response studies, immune assay peptides (ASPs), were synthesized by SPPS and purified by reverse-phase HPLC. Preparative HPLC fractions that met purity and identity criteria based on UPLC-UV/MS analysis were pooled and dried using parallel centrifugal evaporators or lyophilizers. ASPs were 20 aa and overlapped by at least 9 aa to cover the long peptide sequence or were 8–12 aa and predicted to bind class I (ref. ^12^).

#### Personalized ctDNA assay

Design and application of a tumor-informed ctDNA (multiplex polymerase chain reaction (PCR), next-generation sequencing) assay were conducted by Natera with blinding to clinical data. Paired tumor and peripheral blood WES data were used to identify and design the Signatera assay with 16 tumor-specific somatic single-nucleotide variants (SNVs) for each patient. For the Signatera 16-plex assay, SNVs present in the tumor but absent in the germline were used to select the 16 targets, with prioritization based on multiple factors, including the observed variant allele frequency in the tumor tissue and the associated background noise profile in the plasma. The multiplexed targeted PCR was conducted followed by amplicon deep sequencing on an Illumina platform. Variant allele frequencies were determined for each of the target mutations. The plasma sample was considered ctDNA^+^ on observing at least two variants. Absolute ctDNA levels were then calculated for ctDNA^+^ plasma samples (mean tumor molecules (MTM) per milliliter) by normalizing variant allele frequencies observed by the plasma volume used for each sample. As described previously, MTM per milliliter was calculated from all tested targets, including undetected targets^77^.

### Flow cytometry staining

#### MHC class I/II protein expression, purification and peptide exchange

MHC monomers were generated to enable tetramer generation and staining for assessing neoantigen specificity of responses. MHC class I (MHC-I) and MHC class II (MHC-II) monomeric proteins were expressed, purified and peptide exchanged per methods highlighted in Ott et al.^37^ and Vyasamneni et al.^78^, respectively.

#### Tetramer generation

The combinatorial coding multimer approach^79^ was applied, in which biotinylated peptide–MHC monomers (pMHCs) were tetramerized using fluorochrome-conjugated streptavidin, and each pMHC tetramer was assigned a two-color code. To this end, peptide-exchanged alleles were incubated with streptavidin on ice for 30 min in the dark. Biotin was added to block any unoccupied sites on the streptavidin–fluorophore. The reaction was spun down at 2,608*g* (4 °C) for 10 min to remove any aggregates. The tetramer containing supernatants were used for making a pooled tetramer mix.

#### Tetramer staining

PBMCs or DP cells were thawed, counted and treated with 0.025 U μl^−1^ Benzonase and 50 nM dasatinib for 20 min at 37 °C in AIM V (Thermo Fisher Scientific) or X-VIVO (Lonza) media. PBMCs were centrifuged at 458*g* for 5 min, washed once with fluorescence-activated cell sorting (FACS) buffer (1× PBS + 0.5% BSA) supplemented with 50 nM dasatinib and plated in a 96-well plate (up to 3 × 10^6^ cells per well). Cells were resuspended in a pooled tetramer mix made in PBS. For each epitope, tetramers with two different fluorophores were generated, such that a dual color code could be assigned. Cells were washed and stained with tetramers at 37 °C (pMHC-I for 15 min or pMHC-II for 1 h). After tetramer staining, cells were stained with antibodies recognizing surface or intracellular antigens. A detailed example for the gating strategy is presented in Supplementary Fig. 8.

#### Surface and intracellular staining

Staining with surface antibodies was carried out at 4 °C for 30 min. Cells were washed and resuspended in FACS buffer at 4 °C. For panels that did not have intracellular staining, cells were then run on a BD LSRFortessa or a BD FACSymphony A5 instrument. For panels with intracellular markers, cells were fixed and permeabilized with Fixation and Permeabilization Solution (BD Biosciences) for 20 min at room temperature and subsequently stained with antibodies against intracellular proteins at 4 °C for 30 min. Cells were washed and resuspended in FACS buffer and kept at 4 °C until acquisition on the flow cytometers, using FlowJo software (versions 8–10).

To characterize neoantigen-specific responses in the DP, tetramer–cell complexes were gated as follows: live/dead marker^−^, CD14^−^/CD16^−^/CD19^−^/CD4^−^, CD8^+^, tetramer two-color code^+^. To characterize neoantigen-specific responses in peripheral analysis, tetramer–cell complexes were gated as follows: live/dead marker^−^, CD14^−^/CD16^−^/CD19^−^, CD3^+^/CD56^−^, CD4^+^ or CD8^+^, tetramer two-color code^+^.

Corresponding flow panels: DP class I tetramer panel, DP cellular subset characterization panel, peripheral class I/II tetramer panels (Supplementary Data 4).

#### Phenotypic analysis

Differences in median fluorescence intensity (MFI) between tet^+^ and tet^−^ CD8^+^/CD4^+^ T cells were calculated through ‘Transformed Distance in MFI’ (TD_MFI_), which leverages the following calculation: TD_MFI_ = 1/(ln 10) Arcsinh (MFI + /CF) – 1/(ln 10) Arcsinh (MFI − /CF), with a universal cofactor (CF) value of 150. This calculation was adapted from Norton et al.^80^ and Azad et al.^81^.

Corresponding flow panels: peripheral class I/II phenotypic panels (Supplementary Data 4).

### Recall response assay

#### Experimental setup

To identify functional responses, cells were tested in an overnight co-culture, also referred to as recall assay or recall response assay. For DP characterization, DP cells were co-cultured with peptide-loaded DCs. For post-infusion analysis, patient PBMCs were co-cultured with 2 µM peptide(s), which were loaded onto endogenous APCs. Anti-CD107a antibody was added 5 h and GolgiStop/Plug (both BD Biosciences) was added 4 h before the end of co-culture. Subsequently, cells were stained and stored in FACS buffer at 4 °C until acquisition on a BD LSRFortessa or a BD FACSymphony A5 instrument.

#### Functional readouts

To characterize neoantigen-specific responses in the DP, a functional CD4^+^ T cell response was defined by the increased expression of IFNγ and/or TNF in the presence of target antigen compared to non-peptide-loaded DCs. In the flow cytometric readout, cells of interest were gated as follows: live/dead marker^−^, CD14^−^/CD16^−^/CD19^−^/CD3^+^, TagIT violet/CFSE barcodes, CD4^+^. Subsequently, production of IFNγ and/or TNF by the CD4^+^ T cells was assessed. Identified CD4^+^ T cell responses and tet^+^ CD8^+^ responses were further characterized for mutant specificity and polyfunctionality based on a similar functional readout measuring production of IFNγ and/or TNF and/or CD107a.

For post-infusion analysis, CD4^+^ and/or CD8^+^ T cell responses were defined as functional if the frequency of CD107a, IFNγ and/or TNF on HLA-DR^+^ CD4^+^/CD8^+^ T cells stimulated with peptide was 1.5-fold change or greater than DMSO, with a minimum of 50 events in the recall condition.

Corresponding flow panels: peripheral recall panel, DP recall panel (Supplementary Data 4).

### Cytotoxicity assay

Neoantigen-expressing tumor lines were generated by transducing A375 tumor cells (American Type Culture Collection (ATCC)) with DNA encoding for the HLA sequence, neoantigen epitope sequence or wild-type epitope sequence from GeneWiz. The HLA sequences were derived from the Immuno Polymorphism Database (IPD) (https://www.ebi.ac.uk/ipd/) and mutant or wild-type protein sequences from the WES data. SnapGene (versions 4.2, 4.3 and 5.0) was used to design the vectors. The transduced sequence included the flanking region, such that the epitope could be presented only if the antigen presentation machinery of the cell was capable of processing the sequence. Separately, for a subset of the specificities tested, A375 tumor cells with the HLA of interest were exogenously loaded with the peptide of interest.

The transduced tumor cells were then co-cultured with NEO-STIM-induced T cells for 6 h. Subsequently, the tumor and T cells were harvested and stained for surface antibodies and intracellular antibody against caspase 3. Tumor cells were identified through GFP expression when analyzed by flow cytometry. Cytotoxicity was measured through upregulation of active caspase 3 (an early apoptotic marker) on tumor cells.

Corresponding flow panel: DPC cytotox (Supplementary Data 4).

### Functional avidity assay

Functional avidity of neoantigen-specific TCR clones was determined using an NFAT reporter Jurkat model. RNA encoding for the alpha and beta chains of the identified TCRs was generated by in vitro transcription (IVT) of alpha and beta chain DNA constructed through overlapping PCR of eBlock DNA components. Jurkat T cells (ATCC) engineered with an NFAT luciferase reporter to allow for luminescent detection of T cell activation were electroporated with the respective TCRα and TCRβ chain RNA and cultured overnight to allow for TCR expression. Transfection efficiency was evaluated by flow cytometry with an antibody against TCRβ chain. A375 melanoma cells (ATCC), previously transduced with the neoantigen HLA restriction, were plated in a 384-well plate format and then loaded for 2 h with mutant and wild-type (where available) peptides at concentrations ranging from 10 pM to 10 µM. Peptide-loaded A375 cells were then washed with media to remove unbound peptide before co-culture with Jurkat cells. TCR-expressing Jurkat cells and peptide-loaded A375 cells were co-cultured at a 1:1 effector-to-target ratio. PHA-L, a non-specific TCR activator, was used as a positive control to ensure TCR functionality. After overnight co-culture, equal volume of Bio-Glo reagent (Promega) containing the luciferase substrate was added and incubated at room temperature for 15 min. Luminescence was determined on a plate reader. NFAT activation per condition was determined by normalizing luminescence to a negative control reaction containing no peptide. EC_50_ of TCR avidity was determined by graphing NFAT activation against peptide concentration loaded using an [Agonist] versus response (three parameter) fit. In cases where NFAT activation reached saturation, EC_50_ values were determined. In cases where dose–response curves did not reach saturation, no true EC_50_ could be calculated. Estimates of EC_50_ values were reported based on fit curve analysis for cases, where two peptide concentrations showed significant NFAT activation. No EC_50_ is reported for cases where a single concentration showed significant NFAT activation. We also plotted the minimum peptide concentration for NFAT activation, because this is independent of curve saturation. The number of neoantigens tested as well as the number of T cell clones tested per patient are listed in Extended Data Fig. 4c.

Corresponding flow panel: DP TCR avidity quality control (QC) panel (Supplementary Data 4).

### Next-generation sequencing and data analysis

#### RNA extraction from DP, tumors and PBMCs

For formalin-fixed, paraffin-embedded (FFPE) tumor blocks, 2–4 cuts of 20-µm thickness each were deparaffinized using heptane, and RNA was extracted using an AllPrep DNA/RNA FFPE Kit (Qiagen) according to the manufacturer’s instructions. For fresh snap-frozen resection or biopsy tumor samples, RNA was extracted using an RNeasy kit (Qiagen).

For PBMCs and DP, cells were thawed and subjected to negative selection using a Pan T Cell Isolation Kit, Human (Miltenyi Biotec). CD3^+^ T cells were counted, centrifuged for 15 min at 300*g* and then flash frozen and stored as dry pellets at −80 °C. RNA isolation was performed using an RNeasy Plus Micro Kit (Qiagen) on a QIAcube (Qiagen) using the protocol ‘Purification of total RNA using gDNA Eliminator and RNeasy MinElute spin columns’. RNA concentration was measured using a Qubit RNA HS Assay Kit (Thermo Fisher Scientific). Eluted RNA was stored at −80 °C. Upon every RNA extraction step, the quality and integrity of the nucleic acids were evaluated by the RNA integrity numbers (RINs) and DV200 values using a 2100 Bioanalyzer system (Agilent Technologies) before library preparation and sequencing. For RNA extracted from FFPE samples, the RIN score cutoff was ≥2, and, for RNA extracted from fresh-frozen tumors or primary T cells, the cutoff was ≥6.

#### TCR sequencing and phenotype characterization

##### TCR library preparation and bulk TCR-seq

TCRβ libraries were prepared from isolated RNA derived from the snap-frozen T cell pellets (1 million pan T cells each) or tumor at the iRepertoire headquarters. Amp-RepSeq+ (unique molecular identifier (UMI) incorporated) workflow was used for all tumor samples plus PBMC and DP samples from patient NAC05; Amp-RepSeq (non-UMI) workflow was used for PBMC and DP samples from all other patients according to iRepertoire’s proprietary workflow protocol. Libraries were sequenced using a NextSeq Reagent Kit v2 300 cycles (Illumina) at the iRepertoire headquarters according to the manufacturer’s protocol. Throughout the study, the number of samples per pool was designed to maintain equal sequencing depths across samples, allocating target reads of 8 million for peripheral T cells and 1 million reads for tumor biopsies.

##### TCR repertoire generation

TCR repertoires were generated by applying MiXCR 4.3.2 software on the paired-end raw sequencing FASTQ files. Samples sequenced using Amp-RepSeq+ were processed using the preset ‘generic-tcr-amplicon-umi’, with parameters including the species specifications (Human, hsa), starting material (rna), UMI tag pattern (^(UMI:N{10})N{8}(R1:*)\^(R2:*)), plus two alignment flags (–floating-left-alignment-boundary and –floating-right-alignment-boundary C). Samples sequenced with Amp-RepSeq were processed using the preset ‘irepertoire-human-rna-tcr-sr’ with default parameters. TCRβ CDR3 clonotypes were filtered by removal of non-functional sequences (out-of-frame sequences, those containing stop codons or those of length ≤5 aa). Clonal frequency was calculated based on the read count for each clone out of the total read count (non-UMI) or the UMI for each clone out of the total UMI (UMI)^82,83^.

#### Single-cell RNA-seq of neoantigen-specific CD8^+^ T cells using MHC class I tetramer staining

Neoantigen-specific CD8^+^ T cells were activated as described in the ‘Recall response assay’ subsection using peptide-loaded DCs and then sorted and sequenced as described below.

##### Cell sorting using tetramer technology

MHC class I tetramers were prepared as described above and incubated with T cells as outlined in the ‘Tetramer staining’ subsection. After staining, a mix of 34 TotalSeq-C antibodies (BioLegend) was added. Cells were simultaneously labeled with FITC anti-human CD4 (BD Biosciences), FITC anti-human CD14 (BD Biosciences), FITC anti-human CD16 (BD Biosciences), FITC anti-human CD19 (BD Biosciences), BV711 anti-human CD3 (BD Biosciences) and AF700 anti-human CD8 (BioLegend) antibodies and LIVE/DEAD Fixable Near-IR Dead Cell Stain Kit (Thermo Fisher Scientific). After 30 min of incubation at 4 °C, cells were washed twice in PBS with 0.5% w/v BSA and sorted using a FACSAria cell sorter (BD Bioscience). For DP samples, two different CD8^+^ T cell populations were sorted: tet^−^ CD8^+^ (live/dead marker^−^, CD4^−^CD14^−^CD16^−^CD19^−^, CD3^+^, CD8^+^, tet^−^) and tet^+^ CD8^+^ (live/dead marker^−^, CD4^−^/CD14^−^/CD16^−^/CD19^−^, CD3^+^, CD8^+^, tet^+^).

Corresponding flow panel: DP sequencing sort panel (Supplementary Data 4).

##### 10x scGEX/VDJ/CITE-seq

During sorting, labeled cells were collected in PBS, supplemented with 2% FBS and submitted to subsequent single-cell RNA sequencing (scRNA-seq) processes. Sample processing for single-cell gene expression (GEX), TCR V(D)J clonotypes (VDJ) and cell surface protein expression (CITE) libraries was performed using Chromium Next Gel Beads-in-Emulsions (GEM) Single-Cell 5′ Reagent Kits v2 (Dual Index; 10x Genomics) following the manufacturer’s protocol. In brief, sorted tet^+^ and tet^−^ CD8^+^ T cell suspensions were partitioned into nanoliter-scale GEMs using a Chromium Controller (10x Genomics). Subsequent Gel Bead-in-Emulsion reverse transcription (GEM-RT) reaction and post clean-up and cDNA amplification were carried out to generate cDNAs for scRNA-seq libraries. Constructing cDNA libraries for GEX (Library Construction Kit), VDJ (Chromium Single-Cell Human TCR Amplification Kit) and CITE (5′ Feature Barcode Kit) was performed based on the manufacturer’s protocols. All sequencing library quality controls were performed by using Bioanalyzer High Sensitivity DNA Chip Kits with a 2100 Bioanalyzer (Agilent Technologies). Sequencing libraries were quantified using KAPA Library Quantification Kit, Complete Kit, and LightCycler 480 (Roche).

All libraries were incorporated with Sample Index sequences (Dual Index Kit TN Set A for CITE libraries and Dual Index Kit TT Set A for VDJ and GEX libraries) and sequenced on a MiSeq (MiSeq Reagent Kit v2 (300 cycles)) or a NovaSeq S1/2/4 platform (Illumina) depending on sequencing depth. The sequencing parameters were as follows: Read 1 of 26 cycles, i7 Index of 10 cycles, i5 Index of 10 cycles and Read 2 of 90 cycles.

##### scGEX/VDJ/CITE-seq data analysis

Cell Ranger version 6.0.1 (10x Genomics) was used to align raw sequencing data with the ‘multi’ option. The RNA library was aligned on the GRCh38-2020-A genome. The TCR library was aligned on the vdj_GRCh38_alts_ensembl-7.1.0 assembly. The CITE library was aligned on a custom panel of antibodies (Supplementary Data 4, ‘DPC sequencing’). The Python package Scanpy 1.9.1 (ref. ^84^) was used for downstream analysis of data processed with Cell Ranger.

Filtering was performed as follows. We removed cells that had (1) more than 10% mitochondrial gene content; (2) fewer than 1,000 UMIs from RNA; (3) fewer than 300 genes from RNA; (4) fewer than 10 features detected from CITE; or (5) fewer than 100 UMIs (NAC01, NAC02, NAC03, NAC08 and NAC09) or fewer than 500 UMIs (NAC04, NAC05, NAC06 and NAC07) for the CITE library, where the threshold was determined based on the observed distribution of CITE counts. We filtered to include only protein-coding genes, as annotated in GENCODE Basic 42, that were expressed in at least 0.1% of all cells.

RNA libraries were log_2_ normalized following library size normalization with a scale factor of 10^4^; these values were used for visualization of gene expression and identification of highly variable genes. The 4,000 most variable genes were detected by the sc.pp.highly_variable_genes function (flavor = Seurat). These 4,000 genes were overlaid with the 10x Genomics Immunological panel (1,056 genes) to de-noise the list.

Downstream processing was performed on each patient individually to limit batch and donor effects. For integration across samples within each patient, data were normalized as follows. RNA libraries were natural log normalized following library size normalization with a scale factor of 10^4^ and then scaled with the sc.pp.scale function using a maximum value of 10. Total counts were regressed out using the sc.pp.regress_out function. CITE libraries were CLR normalized and then scaled with the sc.pp.scale function using a maximum value of 10. Total counts were regressed out using the sc.pp.regress_out function. Principal component analysis (PCA) was performed with the sc.tl.pca function, and dimensionality reduction was performed using multi-omics factor analysis (MOFA) using the function mu.tl.mofa with 10 factors after restricting to highly variable genes as defined above^85,86^. To create uniform manifold approximation and projection (UMAP) plots, nearest neighbors were found using the sc.pp.neighbors function on the 10 factors from MOFA, and then UMAP dimensionality reduction was performed with sc.tl.umap to obtain a two-dimensional representation of the cell states.

For clustering, we used the Scanpy sc.tl.leiden function. Clusters were assigned phenotypes using RNA and CITE marker genes. In brief, proliferating T cells were defined by expression of *MKI67* (RNA), memory T cells by expression of IL-7RA (CITE), effector T cells by expression of 4-1BB (CITE) and exhausted-like T cells by expression of PD-1 (CITE) in the absence of IL-7R or 4-1BB. In cases where multiple clusters met these broad criteria, subclusters were named using top genes from each subcluster based on differential expression.

#### Identification of neoantigen-specific clonotypes

For each tetramer-sorted sample, we identified antigen-specific clonotypes as those representing at least 0.5% of all cells in the sample, with a minimum of two cells. We removed clonotypes representing likely bystander contamination as follows. For patients where bystander cells from DP were sequenced (NAC01, NAC02, NAC03, NAC05, NAC06, NAC07, NAC08 and NAC09), we excluded clonotypes with significant presence in bystander samples (>10% of cells with that clonotype). For one patient where bystander cells were not sequenced (NAC04), we removed one clonotype that was present in high frequency in pre-treatment PBMCs (>1%) but low frequency in tet^+^ cells (0.5%).

#### Validation of cell type labels for phenotypes

Published gene signatures related to T cell phenotypes with an emphasis on CAR-T cell persistence and dysfunction were collected through a literature search. Differential expression was performed on each cell type using the Scanpy function sc.tl.rank_genes_groups. Gene set enrichment analysis (GSEA) was performed on the returned ranked genes lists using the GSEAPy package version 1.1.3 (ref. ^87^) for all published signatures. Gene sets were filtered to remove genes associated with proliferation, using a set of proliferation gene sets collected from the Molecular Signatures Database (MSigDB) hallmark collection^88^, Reactome^89^, KEGG^90^, BioCarta^91^ and the Pathway Interaction Database^92^.

#### Calculation of T cell activation scores

We used a published T cell activation signature from Fuchs et al.^54^ (Supplementary Table 5 (tab ‘TOP50 separators_expression’)) representing the top 50 genes upregulated in tetramer-sorted antigen-specific CD8^+^ T cells after stimulation with cognate antigen (influenza or cytomegalovirus). Each cell was assigned an activation score representing the average z-score for the gene set using log_2_ (library size normalized) expression values.

### Multiplex immunofluorescence staining

To characterize T cell infiltration in histological sections of the tumor, multiplexing immunofluorescence analysis (MultiOmyx) was performed at NeoGenomics Laboratories. Then, 4-μm sections were cut from FFPE tumor blocks. Each FFPE slide was presented to a NeoGenomics pathologist for tissue annotation and selection. A total of 20 relevant tissue areas were selected by the pathologist and used for analysis.

The FFPE slides were baked, deparaffinized with xylene, rehydrated by washes of decreasing ethanol concentration and then processed for antigen retrieval. Samples were then blocked against non-specific binding and stained with DAPI for nucleus identification. Within each staining round, two cyanine dye-labeled (Cy3 and Cy5) antibodies were paired together and recognized two markers. Antibody specificities included CD3, CD4, CD8 and SOX10 (SOX10 as tumor segmentation marker). The staining signal was then imaged and followed by dye inactivation, enabling repeated rounds of staining.

Individual biomarker image files were merged to generate a single image with multiple markers represented, adjusted for brightness and contrast and then pseudo-colored using ImageJ (version 1.53k) software. Brightness and contrast were applied equally across timepoints.

Quantitative image analysis was performed using proprietary deep-learning-based workflows to identify individual cells and perform cell classification for all individual markers. Individual cell classification results were combined to generate co-expression summaries and compute spatial distribution statistics for phenotypes of interest. Analysis was performed according to NeoGenomics optimized protocols^93^.

### Statistical analysis

Statistical analyses of clinical data were performed using SAS Enterprise Guide (version 8.4). All statistical analyses are deemed exploratory; no confirmatory hypothesis testing was performed. All other exploratory analyses were performed in Python language (version 3.9.15) or GraphPad Prism (version 7.01). Unless otherwise specified, *P* values were derived from a two-tailed Student’s *t*-test.

### Reporting summary

Further information on research design is available in the Nature Portfolio Reporting Summary linked to this article.

## Online content

Any methods, additional references, Nature Portfolio reporting summaries, source data, extended data, supplementary information, acknowledgements, peer review information; details of author contributions and competing interests; and statements of data and code availability are available at 10.1038/s41591-024-03418-4.

## Supplementary information

Supplementary Information Supplementary Figs. 1–8 and Supplementary Tables 1–3.

Reporting Summary

Supplementary Data Set 1 List of neoepitopes tested.

Supplementary Data Set 2 Mutant specificity calculations (*P* values).

Supplementary Data Table 3 Characterization of TCRs.

Supplementary Data Table 4 List of antibody clones and flow cytometry panels.

## Figures and Tables

**Extended Data Table 1:**
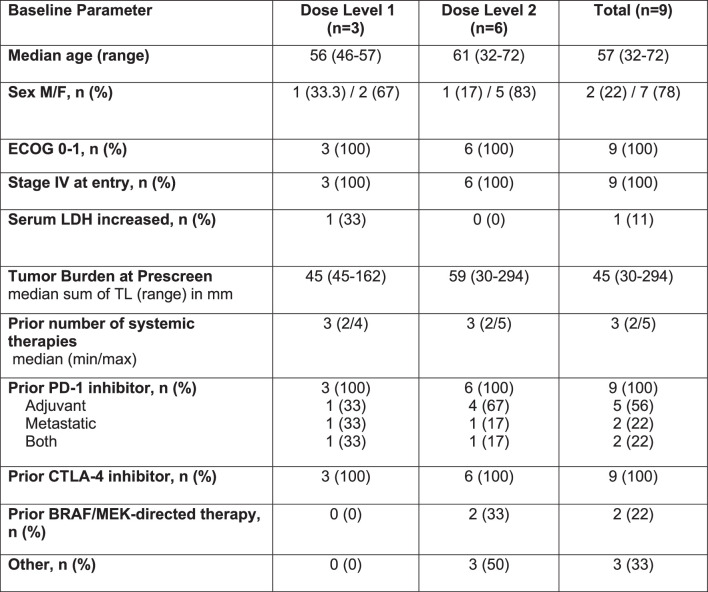
Patient baseline characteristics

Assignment to DLs per intent to treat. See Extended Data Table 2 for actual doses received. Headers are in bold. LDH, lactate dehydrogenase; TL, target lesion.

**Extended Data Table 2:**
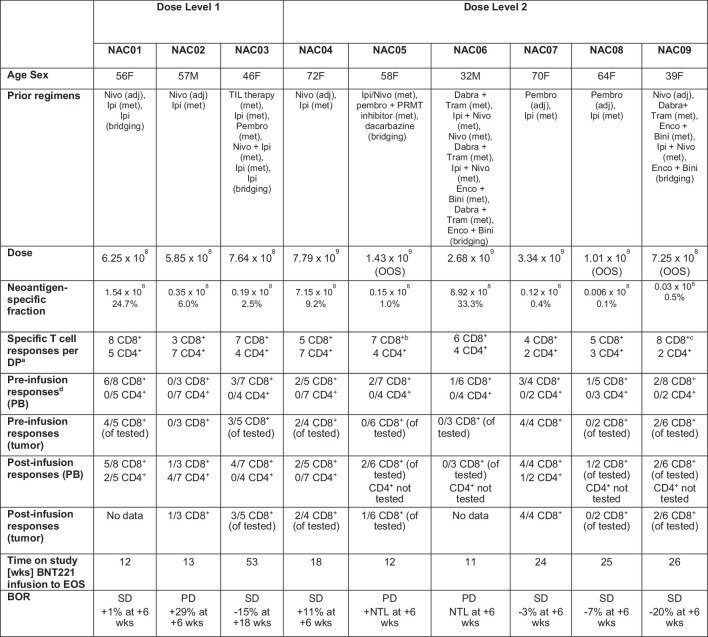
Pre-treatment, treatment and outcome on patient level

^a^Responses reported include those detected in the DP as well as samples taken from each individual culture vessel. A subset of these responses was below the limit of detection in the final DP due to the dilution of cells after pooling.

^b^T cells were induced toward a unique long and short epitope toward DDX42699:M>V in separate individual vessels, leading to a total of seven responses. In the DP, T cells from both responses were quantified together.

^c^One of the responses identified in an individual vessel was not quantified in the DP due to lack of sample availability.

^d^CD8^+^ T cell responses were analyzed by both flow cytometry and bulk TCR-seq. CD4^+^ T cell responses were analyzed by flow cytometry only.

Assignment to DLs per intent to treat. See row 3 for actual doses received.

Ag, antigen; adj, adjuvant; Bini, binimetinib (MEK inhibitor); BOR, best overall response; Dabra, dabrafenib (BRAF inhibitor); Enco, encorafenib (BRAF inhibitor); EOS, end of study; Ipi, ipilimumab (anti-CTLA-4); met, anti-PD-1 treatment given in the metastatic setting; Nivo, nivolumab (anti-PD-1); OOS, out of specification—see ‘Identification of highest tolerable dose’ subsection; PB, peripheral blood; Pembro, pembrolizumab (anti-PD-1); Tram, trametinib (MEK inhibitor); +NTL, emergence of new non-target lesions. Abbreviations for patient IDs: NAC, neoantigen cell dose received; NVD, never dosed. Headers are in bold.

## Data Availability

This trial is currently ongoing. Upon completion of this clinical trial, summary-level results will be made public and shared in line with clinical data-sharing guidelines. Requests for access to aggregated clinical data will be reviewed and approved by the Safety Review Committee on the basis of scientific merit. The datasets generated during the current study and the custom bioinformatics analysis are not publicly available due to proprietary considerations beyond the data that were made available here. All data provided are anonymized to respect the privacy of patients who have participated in the trial, in line with applicable laws and regulations. Data requests pertaining to this paper may be made to the corresponding authors (M.M.v.B., marit.vanbuuren@biontech.us, and J.B.H., j.haanen@nki.nl). Requests will be processed within 16 weeks. NEO-STIM patents are pending in various countries (WO2020227546A1 and WO2023064930A1). Datasets generated in the study include patient-derived single-cell GEX/CITE/VDJ and bulk TCR datasets generated at multiple timepoints. Public datasets and databases used in this study include GRCh38-2020-A genome and vdj_GRCh38_alts_ensembl-7.1.0 assembly (https://www.10xgenomics.com/support/software/cell-ranger/downloads#reference-downloads); 10x genomics Immunological panel 1,056 genes (https://www.10xgenomics.com/support/single-cell-gene-expression/documentation/steps/targeted-gene-expression/human-immunology-panel); Activation score Fuchs et al.^54^, Supplementary Table 5; and Dysfunction score Good et al.^55^. MSigDB hallmark collection^88^, Reactome^89^, KEGG^90^, BioCarta^91^ and the Pathway Interaction Database^92^ were downloaded from https://www.gsea-msigdb.org/gsea/msigdb, and some curation was performed for this paper.

## References

[1] Michielin, O. et al. Cutaneous melanoma: ESMO Clinical Practice Guidelines for diagnosis, treatment and follow-up. *Ann. Oncol.* **30**, 1884–1901 (2019). 31566661 10.1093/annonc/mdz411

[2] Seth, R. et al. Systemic therapy for melanoma: ASCO guideline update. *J. Clin. Oncol.* **41**, 4794–4820 (2023). 37579248 10.1200/JCO.23.01136

[3] Larkin, J. et al. Five-year survival with combined nivolumab and ipilimumab in advanced melanoma. *N. Engl. J. Med.* **381**, 1535–1546 (2019). 31562797 10.1056/NEJMoa1910836

[4] Curti, B. D. & Faries, M. B. Recent advances in the treatment of melanoma. *N. Engl. J. Med.* **384**, 2229–2240 (2021). 34107182 10.1056/NEJMra2034861

[5] Hamid, O. et al. Five-year survival outcomes for patients with advanced melanoma treated with pembrolizumab in KEYNOTE-001. *Ann. Oncol.* **30**, 582–588 (2019). 30715153 10.1093/annonc/mdz011 PMC6503622

[6] Rosenberg, S. A. & Restifo, N. P. Adoptive cell transfer as personalized immunotherapy for human cancer. *Science* **348**, 62–68 (2015). 25838374 10.1126/science.aaa4967 PMC6295668

[7] Waldman, A. D., Fritz, J. M. & Lenardo, M. J. A guide to cancer immunotherapy: from T cell basic science to clinical practice. *Nat. Rev. Immunol.* **20**, 651–668 (2020). 32433532 10.1038/s41577-020-0306-5 PMC7238960

[8] Hacohen, N., Fritsch, E. F., Carter, T. A., Lander, E. S. & Wu, C. J. Getting personal with neoantigen-based therapeutic cancer vaccines. *Cancer Immunol. Res.* **1**, 11–15 (2013). 24777245 10.1158/2326-6066.CIR-13-0022 PMC4033902

[9] Hu, Z. et al. Personal neoantigen vaccines induce persistent memory T cell responses and epitope spreading in patients with melanoma. *Nat. Med.* **27**, 515–525 (2021). 33479501 10.1038/s41591-020-01206-4 PMC8273876

[10] Jamal-Hanjani, M., Quezada, S. A., Larkin, J. & Swanton, C. Translational implications of tumor heterogeneity. *Clin. Cancer Res.* **21**, 1258–1266 (2015). 25770293 10.1158/1078-0432.CCR-14-1429 PMC4374162

[11] Keskin, D. B. et al. Neoantigen vaccine generates intratumoral T cell responses in phase Ib glioblastoma trial. *Nature* **565**, 234–239 (2019). 30568305 10.1038/s41586-018-0792-9 PMC6546179

[12] Ott, P. A. et al. An immunogenic personal neoantigen vaccine for patients with melanoma. *Nature* **547**, 217–221 (2017). 28678778 10.1038/nature22991 PMC5577644

[13] Rohaan, M. W. et al. Tumor-infiltrating lymphocyte therapy or ipilimumab in advanced melanoma. *N. Engl. J. Med.* **387**, 2113–2125 (2022). 36477031 10.1056/NEJMoa2210233

[14] Chesney, J. et al. Efficacy and safety of lifileucel, a one-time autologous tumor-infiltrating lymphocyte (TIL) cell therapy, in patients with advanced melanoma after progression on immune checkpoint inhibitors and targeted therapies: pooled analysis of consecutive cohorts of the C-144-01 study. *J. Immunother. Cancer* **10**, e005755 (2022). 36600653 10.1136/jitc-2022-005755 PMC9748991

[15] Sarnaik, A. A. et al. Lifileucel, a tumor-infiltrating lymphocyte therapy, in metastatic melanoma. *J. Clin. Oncol.* **39**, 2656–2666 (2021). 33979178 10.1200/JCO.21.00612 PMC8376325

[16] US Food & Drug Administration. AMTAGVI. https://www.fda.gov/vaccines-blood-biologics/approved-blood-products/amtagvi (2024).

[17] Klobuch, S., Seijkens, T. T. P., Schumacher, T. N. & Haanen, J. Tumour−infiltrating lymphocyte therapy for patients with advanced stage melanoma. *Nat. Rev. Clin. Oncol.* **21**, 173–184 (2024). 38191921 10.1038/s41571-023-00848-w

[18] Woroniecka, K. et al. T-cell exhaustion signatures vary with tumor type and are severe in glioblastoma. *Clin. Cancer Res.* **24**, 4175–4186 (2018). 29437767 10.1158/1078-0432.CCR-17-1846 PMC6081269

[19] Yossef, R. et al. Phenotypic signatures of circulating neoantigen-reactive CD8^+^ T cells in patients with metastatic cancers. *Cancer Cell* **41**, 2154–2165 (2023). 38039963 10.1016/j.ccell.2023.11.005 PMC10843665

[20] Bianchi, V., Harari, A. & Coukos, G. Neoantigen-specific adoptive cell therapies for cancer: making T-cell products more personal. *Front. Immunol.* **11**, 1215 (2020). 32695101 10.3389/fimmu.2020.01215 PMC7333784

[21] van den Berg, J. H. et al. Tumor infiltrating lymphocytes (TIL) therapy in metastatic melanoma: boosting of neoantigen-specific T cell reactivity and long-term follow-up. *J. Immunother. Cancer* **8**, e000848 (2020). 32753545 10.1136/jitc-2020-000848 PMC7406109

[22] Johnson, L. A. et al. Gene therapy with human and mouse T-cell receptors mediates cancer regression and targets normal tissues expressing cognate antigen. *Blood* **114**, 535–546 (2009). 19451549 10.1182/blood-2009-03-211714 PMC2929689

[23] Robbins, P. F. et al. A pilot trial using lymphocytes genetically engineered with an NY-ESO-1-reactive T-cell receptor: long-term follow-up and correlates with response. *Clin. Cancer Res.* **21**, 1019–1027 (2015). 25538264 10.1158/1078-0432.CCR-14-2708 PMC4361810

[24] Robbins, P. F. et al. Tumor regression in patients with metastatic synovial cell sarcoma and melanoma using genetically engineered lymphocytes reactive with NY-ESO-1. *J. Clin. Oncol.* **29**, 917–924 (2011). 21282551 10.1200/JCO.2010.32.2537 PMC3068063

[25] Hong, D. S. et al. Autologous T cell therapy for MAGE-A4^+^ solid cancers in HLA-A*02^+^ patients: a phase 1 trial. *Nat. Med.* **29**, 104–114 (2023). 36624315 10.1038/s41591-022-02128-z PMC9873554

[26] Liu, Y. et al. TCR-T immunotherapy: the challenges and solutions. *Front. Oncol.* **11**, 794183 (2021). 35145905 10.3389/fonc.2021.794183 PMC8822241

[27] Foy, S. P. et al. Non-viral precision T cell receptor replacement for personalized cell therapy. *Nature* **615**, 687–696 (2023). 36356599 10.1038/s41586-022-05531-1 PMC9768791

[28] Pang, Z. et al. Neoantigen-targeted TCR-engineered T cell immunotherapy: current advances and challenges. *Biomark. Res.* **11**, 104 (2023). 38037114 10.1186/s40364-023-00534-0 PMC10690996

[29] Leidner, R. et al. Neoantigen T-cell receptor gene therapy in pancreatic cancer. *N. Engl. J. Med.* **386**, 2112–2119 (2022). 35648703 10.1056/NEJMoa2119662 PMC9531755

[30] Morelli, M. et al. Safety and efficacy of Sleeping Beauty TCR-T cells targeting shared *KRAS* and *TP53* mutations expressed by solid tumors in first-in-human phase 1 study. *J. Clin. Oncol.*10.1200/JCO.2023.41.16_suppl.2547 (2023).

[31] Kaluza, K. M. et al. Adoptive T cell therapy promotes the emergence of genomically altered tumor escape variants. *Int. J. Cancer* **131**, 844–854 (2012). 21935923 10.1002/ijc.26447 PMC3903054

[32] Olivier, T., Haslam, A., Tuia, J. & Prasad, V. Eligibility for human leukocyte antigen-based therapeutics by race and ethnicity. *JAMA Netw. Open* **6**, e2338612 (2023). 37883087 10.1001/jamanetworkopen.2023.38612 PMC10603498

[33] Smithy, J. W., Blouin, A., Diamond, L. C. & Postow, M. Ensuring equity in the era of HLA-restricted cancer therapeutics. *J. Immunother. Cancer* **10**, e005600 (2022). 36442912 10.1136/jitc-2022-005600 PMC9710357

[34] Lenkala, D. et al. 153 NEO-PTC-01 (BNT221), an autologous neoantigen-specific T-cell product for adoptive cell therapy of metastatic melanoma. *J. Immunother. Cancer* **8**, A92–A93 (2020).

[35] Abelin, J. G. et al. Defining HLA-II ligand processing and binding rules with mass spectrometry enhances cancer epitope prediction. *Immunity* **51**, 766–779 (2019). 31495665 10.1016/j.immuni.2019.08.012

[36] Abelin, J. G. et al. Mass spectrometry profiling of HLA-associated peptidomes in mono-allelic cells enables more accurate epitope prediction. *Immunity* **46**, 315–326 (2017). 28228285 10.1016/j.immuni.2017.02.007 PMC5405381

[37] Ott, P. A. et al. A phase Ib trial of personalized neoantigen therapy plus anti-PD-1 in patients with advanced melanoma, non-small cell lung cancer, or bladder cancer. *Cell* **183**, 347–362 (2020). 33064988 10.1016/j.cell.2020.08.053

[38] Alexandrov, L. B. et al. Signatures of mutational processes in human cancer. *Nature* **500**, 415–421 (2013). 23945592 10.1038/nature12477 PMC3776390

[39] Li, D. et al. A pilot study of lymphodepletion intensity for peripheral blood mononuclear cell-derived neoantigen-specific CD8 + T cell therapy in patients with advanced solid tumors. *Nat. Commun.* **14**, 3447 (2023). 37301885 10.1038/s41467-023-39225-7 PMC10257664

[40] Singer, A., Adoro, S. & Park, J. H. Lineage fate and intense debate: myths, models and mechanisms of CD4- versus CD8-lineage choice. *Nat. Rev. Immunol.* **8**, 788–801 (2008). 18802443 10.1038/nri2416 PMC2760737

[41] Majzner, R. G. & Mackall, C. L. Tumor antigen escape from CAR T-cell therapy. *Cancer Discov.* **8**, 1219–1226 (2018). 30135176 10.1158/2159-8290.CD-18-0442

[42] Nagarsheth, N. B. et al. TCR-engineered T cells targeting E7 for patients with metastatic HPV-associated epithelial cancers. *Nat. Med.* **27**, 419–425 (2021). 33558725 10.1038/s41591-020-01225-1 PMC9620481

[43] Linnemann, C. et al. High-throughput epitope discovery reveals frequent recognition of neo-antigens by CD4^+^ T cells in human melanoma. *Nat. Med.* **21**, 81–85 (2015). 25531942 10.1038/nm.3773

[44] Tran, E. et al. Cancer immunotherapy based on mutation-specific CD4^+^ T cells in a patient with epithelial cancer. *Science* **344**, 641–645 (2014). 24812403 10.1126/science.1251102 PMC6686185

[45] van Rooij, N. et al. Tumor exome analysis reveals neoantigen-specific T-cell reactivity in an ipilimumab-responsive melanoma. *J. Clin. Oncol.* **31**, e439–e442 (2013). 24043743 10.1200/JCO.2012.47.7521 PMC3836220

[46] George, A. J., Stark, J. & Chan, C. Understanding specificity and sensitivity of T-cell recognition. *Trends Immunol.* **26**, 653–659 (2005). 16236548 10.1016/j.it.2005.09.011

[47] Seder, R. A., Darrah, P. A. & Roederer, M. T-cell quality in memory and protection: implications for vaccine design. *Nat. Rev. Immunol.* **8**, 247–258 (2008). 18323851 10.1038/nri2274

[48] Yuan, J. et al. CTLA-4 blockade enhances polyfunctional NY-ESO-1 specific T cell responses in metastatic melanoma patients with clinical benefit. *Proc. Natl Acad. Sci. USA* **105**, 20410–20415 (2008). 19074257 10.1073/pnas.0810114105 PMC2629307

[49] Krishna, S. et al. Stem-like CD8 T cells mediate response of adoptive cell immunotherapy against human cancer. *Science* **370**, 1328–1334 (2020). 33303615 10.1126/science.abb9847 PMC8883579

[50] Sade-Feldman, M. et al. Defining T cell states associated with response to checkpoint immunotherapy in melanoma. *Cell* **176**, P404 (2019). 10.1016/j.cell.2018.12.034 PMC6647017 30633907

[51] Chen, G. M. et al. Integrative bulk and single-cell profiling of premanufacture T-cell populations reveals factors mediating long-term persistence of CAR T-cell therapy. *Cancer Discov.* **11**, 2186–2199 (2021). 33820778 10.1158/2159-8290.CD-20-1677 PMC8419030

[52] Fraietta, J. A. et al. Determinants of response and resistance to CD19 chimeric antigen receptor (CAR) T cell therapy of chronic lymphocytic leukemia. *Nat. Med.* **24**, 563–571 (2018). 29713085 10.1038/s41591-018-0010-1 PMC6117613

[53] Lu, Y. C. et al. Single-cell transcriptome analysis reveals gene signatures associated with T-cell persistence following adoptive cell therapy. *Cancer Immunol. Res.* **7**, 1824–1836 (2019). 31484655 10.1158/2326-6066.CIR-19-0299 PMC6825592

[54] Fuchs, Y. F. et al. Gene expression-based identification of antigen-responsive CD8^+^ T cells on a single-cell level. *Front. Immunol.* **10**, 2568 (2019). 31781096 10.3389/fimmu.2019.02568 PMC6851025

[55] Good, C. R. et al. An NK-like CAR T cell transition in CAR T cell dysfunction. *Cell* **184**, 6081–6100 (2021). 34861191 10.1016/j.cell.2021.11.016 PMC8827167

[56] Long, A. H. et al. 4-1BB costimulation ameliorates T cell exhaustion induced by tonic signaling of chimeric antigen receptors. *Nat. Med.* **21**, 581–590 (2015). 25939063 10.1038/nm.3838 PMC4458184

[57] Szabo, P. A. et al. Single-cell transcriptomics of human T cells reveals tissue and activation signatures in health and disease. *Nat. Commun.* **10**, 4706 (2019). 31624246 10.1038/s41467-019-12464-3 PMC6797728

[58] Campillo-Davo, D., Flumens, D. & Lion, E. The quest for the best: how TCR affinity, avidity, and functional avidity affect TCR-engineered T-cell antitumor responses. *Cells* **9**, 1720 (2020). 32708366 10.3390/cells9071720 PMC7408146

[59] Wei, F., Cheng, X. X., Xue, J. Z. & Xue, S. A. Emerging strategies in TCR-engineered T cells. *Front. Immunol.* **13**, 850358 (2022). 35432319 10.3389/fimmu.2022.850358 PMC9006933

[60] Parkhurst, M. R. et al. Unique neoantigens arise from somatic mutations in patients with gastrointestinal cancers. *Cancer Discov.* **9**, 1022–1035 (2019). 31164343 10.1158/2159-8290.CD-18-1494 PMC7138461

[61] Rizvi, N. A. et al. Cancer immunology. Mutational landscape determines sensitivity to PD-1 blockade in non-small cell lung cancer. *Science* **348**, 124–128 (2015). 25765070 10.1126/science.aaa1348 PMC4993154

[62] Robbins, P. F. et al. Mining exomic sequencing data to identify mutated antigens recognized by adoptively transferred tumor-reactive T cells. *Nat. Med.* **19**, 747–752 (2013). 23644516 10.1038/nm.3161 PMC3757932

[63] Tran, E. et al. Immunogenicity of somatic mutations in human gastrointestinal cancers. *Science* **350**, 1387–1390 (2015). 26516200 10.1126/science.aad1253 PMC7445892

[64] Daniel, B. et al. Divergent clonal differentiation trajectories of T cell exhaustion. *Nat. Immunol.* **23**, 1614–1627 (2022). 36289450 10.1038/s41590-022-01337-5 PMC11225711

[65] Schmidt, J. et al. Neoantigen-specific CD8 T cells with high structural avidity preferentially reside in and eliminate tumors. *Nat. Commun.* **14**, 3188 (2023). 37280206 10.1038/s41467-023-38946-z PMC10244384

[66] Ayuk, F. A. et al. Axicabtagene ciloleucel in vivo expansion and treatment outcome in aggressive B-cell lymphoma in a real-world setting. *Blood Adv.* **5**, 2523–2527 (2021). 34100900 10.1182/bloodadvances.2020003959 PMC8238487

[67] Hay, K. A. et al. Factors associated with durable EFS in adult B-cell ALL patients achieving MRD-negative CR after CD19 CAR T-cell therapy. *Blood* **133**, 1652–1663 (2019). 30728140 10.1182/blood-2018-11-883710 PMC6460418

[68] Porter, D. L. et al. Chimeric antigen receptor T cells persist and induce sustained remissions in relapsed refractory chronic lymphocytic leukemia. *Sci. Transl. Med.* **7**, 303ra139 (2015). 10.1126/scitranslmed.aac5415 PMC5909068 26333935

[69] Rosenberg, S. A. et al. Durable complete responses in heavily pretreated patients with metastatic melanoma using T-cell transfer immunotherapy. *Clin. Cancer Res.* **17**, 4550–4557 (2011). 21498393 10.1158/1078-0432.CCR-11-0116 PMC3131487

[70] Righi, M. et al. Enhancing CAR T-cell therapy using Fab-based constitutively heterodimeric cytokine receptors. *Cancer Immunol. Res.* **11**, 1203–1221 (2023). 37352396 10.1158/2326-6066.CIR-22-0640 PMC10472109

[71] Doan, A. E. et al. FOXO1 is a master regulator of memory programming in CAR T cells. *Nature* **629**, 211–218 (2024). 38600391 10.1038/s41586-024-07300-8 PMC11062920

[72] Flosbach, M. et al. PTPN2 deficiency enhances programmed T cell expansion and survival capacity of activated T cells. *Cell Rep.* **32**, 107957 (2020). 32726622 10.1016/j.celrep.2020.107957 PMC7408006

[73] Lynn, R. C. et al. c-Jun overexpression in CAR T cells induces exhaustion resistance. *Nature* **576**, 293–300 (2019). 31802004 10.1038/s41586-019-1805-z PMC6944329

[74] Sailer, N. et al. T-cells expressing a highly potent PRAME-specific T-cell receptor in combination with a chimeric PD1-41BB co-stimulatory receptor show a favorable preclinical safety profile and strong anti-tumor reactivity. *Cancers (Basel)* **14**, 1998 (2022). 35454906 10.3390/cancers14081998 PMC9030144

[75] Mackensen, A. et al. CLDN6-specific CAR-T cells plus amplifying RNA vaccine in relapsed or refractory solid tumors: the phase 1 BNT211-01 trial. *Nat. Med.* **29**, 2844–2853 (2023). 37872225 10.1038/s41591-023-02612-0 PMC10667102

[76] Tran, E. et al. T-cell transfer therapy targeting mutant KRAS in cancer. *N. Engl. J. Med.* **375**, 2255–2262 (2016). 27959684 10.1056/NEJMoa1609279 PMC5178827

[77] Bratman, S. V. et al. Personalized circulating tumor DNA analysis as a predictive biomarker in solid tumor patients treated with pembrolizumab. *Nat. Cancer* **1**, 873–881 (2020). 35121950 10.1038/s43018-020-0096-5

[78] Vyasamneni, R. et al. A universal MHCII technology platform to characterize antigen-specific CD4^+^ T cells. *Cell Rep. Methods* **3**, 100388 (2023). 36814840 10.1016/j.crmeth.2022.100388 PMC9939426

[79] Andersen, R. S. et al. Parallel detection of antigen-specific T cell responses by combinatorial encoding of MHC multimers. *Nat. Protoc.* **7**, 891–902 (2012). 22498709 10.1038/nprot.2012.037

[80] Norton, E. C. The inverse hyperbolic sine transformation and retransformed marginal effects. *Stata J.* **22**, 702–712 (2022).

[81] Azad, A., Rajwa, B. & Pothen, A. flowVS: channel-specific variance stabilization in flow cytometry. *BMC Bioinformatics* **17**, 291 (2016). 27465477 10.1186/s12859-016-1083-9 PMC4964071

[82] Jia, Q. et al. Diversity index of mucosal resident T lymphocyte repertoire predicts clinical prognosis in gastric cancer. *Oncoimmunology* **4**, e1001230 (2015). 26137399 10.1080/2162402X.2014.1001230 PMC4485732

[83] Hanson, A. L. et al. Altered repertoire diversity and disease-associated clonal expansions revealed by T cell receptor immunosequencing in ankylosing spondylitis patients. *Arthritis Rheumatol.* **72**, 1289–1302 (2020). 32162785 10.1002/art.41252

[84] Wolf, F. A., Angerer, P. & Theis, F. J. SCANPY: large-scale single-cell gene expression data analysis. *Genome Biol.* **19**, 15 (2018). 29409532 10.1186/s13059-017-1382-0 PMC5802054

[85] Argelaguet, R. et al. MOFA+: a statistical framework for comprehensive integration of multimodal single-cell data. *Genome Biol.* **21**, 111 (2020). 32393329 10.1186/s13059-020-02015-1 PMC7212577

[86] Bredikhin, D., Kats, I. & Stegle, O. MUON: multimodal omics analysis framework. *Genome Biol.* **23**, 42 (2022). 35105358 10.1186/s13059-021-02577-8 PMC8805324

[87] Fang, Z., Liu, X. & Peltz, G. GSEApy: a comprehensive package for performing gene set enrichment analysis in Python. *Bioinformatics* **39**, btac757 (2022). 10.1093/bioinformatics/btac757 PMC9805564 36426870

[88] Liberzon, A. et al. The Molecular Signatures Database (MSigDB) hallmark gene set collection. *Cell Syst.* **1**, 417–425 (2015). 26771021 10.1016/j.cels.2015.12.004 PMC4707969

[89] Vastrik, I. et al. Reactome: a knowledge base of biologic pathways and processes. *Genome Biol.* **8**, R39 (2007). 17367534 10.1186/gb-2007-8-3-r39 PMC1868929

[90] Kanehisa, M. & Goto, S. KEGG: Kyoto Encyclopedia of Genes and Genomes. *Nucleic Acids Res.* **28**, 27–30 (2000). 10592173 10.1093/nar/28.1.27 PMC102409

[91] Nishimura, D. BioCarta. *Biotech. Softw. Internet Rep.* **2**, 117–120 (2001).

[92] Schaefer, C. F. et al. PID: the Pathway Interaction Database. *Nucleic Acids Res.* **37**, D674–D679 (2009). 18832364 10.1093/nar/gkn653 PMC2686461

[93] Gerdes, M. J. et al. Highly multiplexed single-cell analysis of formalin-fixed, paraffin-embedded cancer tissue. *Proc. Natl Acad. Sci. USA* **110**, 11982–11987 (2013). 23818604 10.1073/pnas.1300136110 PMC3718135

