# Publisher Correction: Personalized, autologous neoantigen-specific T cell therapy in metastatic melanoma: a phase 1 trial

**DOI:** 10.1038/s41591-025-03896-0

**Published:** 2025-08-04

**Authors:** Jessica S. W. Borgers, Divya Lenkala, Victoria Kohler, Emily K. Jackson, Matthijs D. Linssen, Sebastian Hymson, Brian McCarthy, Elizabeth O’Reilly Cosgrove, Kristen N. Balogh, Ekaterina Esaulova, Kimberly Starr, Yvonne Ware, Sebastian Klobuch, Tracey Sciuto, Xi Chen, Gauri Mahimkar, Joong Hyuk F. Sheen, Suchitra Ramesh, Sofie Wilgenhof, Johannes V. van Thienen, Karina C. Scheiner, Inge Jedema, Michael Rooney, Jesse Z. Dong, John R. Srouji, Vikram R. Juneja, Christina M. Arieta, Bastiaan Nuijen, Claudia Gottstein, Olivia C. Finney, Kelledy Manson, Cynthia M. Nijenhuis, Richard B. Gaynor, Mark DeMario, John B. Haanen, Marit M. van Buuren

**Affiliations:** 1 Department of Medical Oncology, Netherlands Cancer Institute (NKI), Amsterdam, The Netherlands; 2 BioNTech US, Cambridge, MA USA; 3 BioTherapeutics Unit, Division of Pharmacy and Pharmacology, Netherlands Cancer Institute (NKI), Amsterdam, The Netherlands; 4 BioNTech SE, Mainz, Germany; 5 Division of Molecular Oncology and Immunology, Netherlands Cancer Institute (NKI), Amsterdam, The Netherlands

**Keywords:** Drug development, Tumour immunology

Correction to: *Nature Medicine* https://doi.org/10.1038/s41591-024-03418-4, published online 3 January 2025.

In the version of the article initially published, the *y*-axis label in Fig. 3f was incorrect and has now been amended to “% Cas3^+^ of live target cells”. Additionally, this article was originally published under Creative Commons Attribution 4.0 International license, CC-BY-NC-ND, © The Author(s). It is now available under a Creative Commons Attribution 4.0 International license, CC-BY, © The Author(s). These corrections have been made to the HTML and PDF versions of the article.

