# Supplementary material for: Personalized, autologous neoantigen-specific T cell therapy in metastatic melanoma: a phase 1 trial
Source: Nat Med. 2025 Jan 3;31(3):881–93. doi: 10.1038/s41591-024-03418-4 (PMC11922764; doi:10.1038/s41591-024-03418-4)
Supplement: Supplementary file 1 — Supplementary Figs. 1–8 and Supplementary Tables 1–3. [file 41591_2024_3418_MOESM1_ESM.pdf]

nature medicine

Supplementary information

https://doi.org/10.1038/s41591-024-03418-4

Personalized, autologous neoantigen-specific T cell therapy in metastatic melanoma: a phase 1 trial

In the format provided by the authors and unedited

List of Supplementary Information

Supplementary Figure 1	Measurement of ctDNA in the peripheral blood.
Supplementary Figure 2	Tetramer staining of neoantigen-specific T cells in the drug product.
Supplementary Figure 3	Gating strategy for specificity experiment.
Supplementary Figure 4	Induced T cell responses are mutant-reactive.
Supplementary Figure 5	Validation of cell type labels.
Supplementary Figure 6	Phenotype determination of DP cells by scGEX/VDJ/CITE-seq.
Supplementary Figure 7	Phenotype determination of neoantigen-specific T cells post-infusion.
Supplementary Figure 8	Example for Flow Cytometry gating strategy.
Supplementary Table 1	Overall study-related AEs.
Supplementary Table 2	Planned changes during trial conduct.
Supplementary Data S1	List of neoepitopes tested (Excel).
Supplementary Data S2	Mutant specificity calculations (*p*-values, Excel).
Supplementary Data S3	Characterization of T cell receptors (Excel).
Supplementary Data S4	List of antibody clones and flow cytometry panels (Excel).

Supplementary Figures

Supplementary Figure 1:

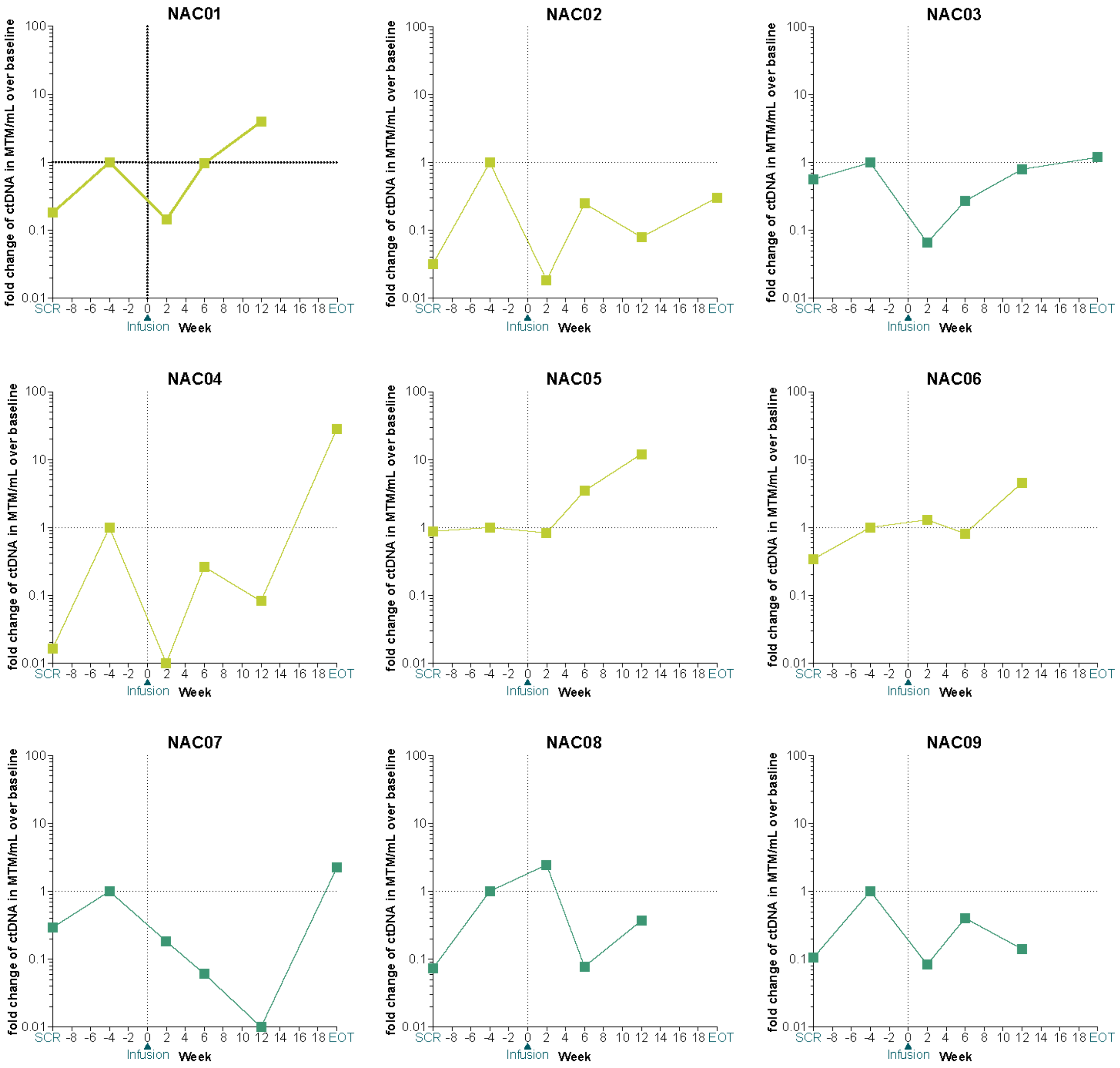

Supplementary Figure 1: Measurement of ctDNA in the peripheral blood.

ctDNA measurements for nine patients from pre- and post-infusion, as measured by the fold change in mean tumor molecules (MTM) per mL of blood; dark green symbols represent patients who experienced tumor regression post-infusion, and light green symbols represent patients who did not experience tumor regression post-infusion. Samples below the limit of detection for the assay are shown at 0.01. Values from week -4 were set as baseline. Abbreviations for patient IDs: NAC: neoantigen cell dose received.

Supplementary Figure 2:

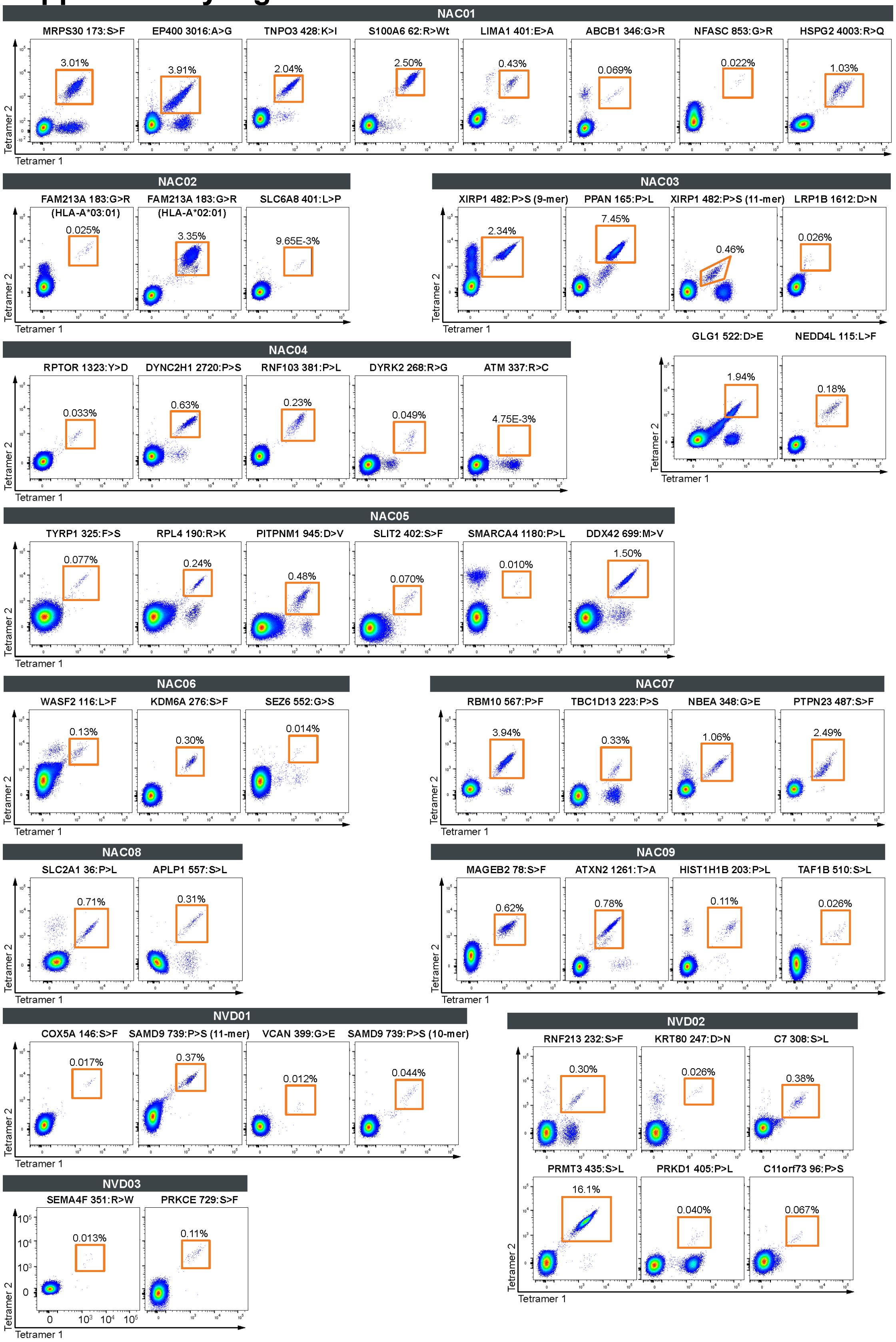

Supplementary Figure 2: Tetramer staining of neoantigen-specific cells in the drug product.

Scatter plots from tetramer-based flow cytometry, showing neoantigen-specific $CD8^+$ T cell responses in drug products from all enrolled patients. Percentages in the boxes are neoantigen-specific T cells out of all $CD8^+$ cells. Tetramer 1, 2: Neoantigen-specific tetramers labeled with different fluorochromes. Fluorophores used were as follows: NAC01 tetramer 1 (left to right [L to R]): PE, PE, PE, BV421, BV421, PE, BV421, BV421; NAC01 tetramer 2 (L to R): BV421, BV650, BUV396, BV650, BUV396, BV421, BV605, BV605: NAC02 tetramer 1: PE; NAC02 tetramer 2 (L to R): BUV396, BV421, BV650; NAC03 tetramer 1 top row (L to R): BV421, PE, PE, BV421; NAC03 tetramer 1 bottom row (L to R): BUV396, PE; NAC03 tetramer 2 top row (L to R): BUV396, BUV396, BV605, BUV396; NAC03 tetramer 2 bottom row (L to R): BV650, BV421; NAC04 tetramer 1 (L to R): BV421, PE, PE, PE, PE; NAC04 tetramer 2 (L to R): BV605, BUV396, BUV396, BV421, BV421; NAC05 tetramer 1: PE; NAC05 tetramer 2 (L toR): BUV396, BUV396, BV650, BV650, BUV396, BUV396; NAC06 tetramer 1: PE; NAC06 tetramer 2 (L to R): BUV396, BV650, BUV396; NAC07 tetramer 1: PE; NAC07 tetramer 2 (L tor R): BUV396, BV650, BUV396, BUV396; NAC08 tetramer 1 (L to R): BUV396, BV605; NAC08 tetramer 2: (L to R): BV605, BV650; NAC09 tetramer 1: PE; NAC09 tetramer 2 (L to R): BUV396, BV421, BV421, BV605; NVD01 tetramer 1 (L to R): BV421, BV421, PE, PE; NVD01 tetramer 2 (L to R): BV605, BUV396, BV605, BV650; NVD02 tetramer 1 top row (L to R): PE, BV421, PE: NVD02 tetramer 1 bottom row (L to R): PE, PE, BV421; NVD02 tetramer 2 top row (L to R): BV650, BV650, BUV396; NVD02 tetramer 2 bottom row (L to R): BV650, BV421, BV605; NVD03 tetramer 1: PE; NVD03 tetramer 2: (L to R): BUV396, BV650. In order to avoid the detection of false positives, tetramers that use different color combinations were used to confirm responses with an additional sample (data not shown). Abbreviations for patient IDs: NAC: neoantigen cell dose received; NVD: never dosed.

Supplementary Figure 3:

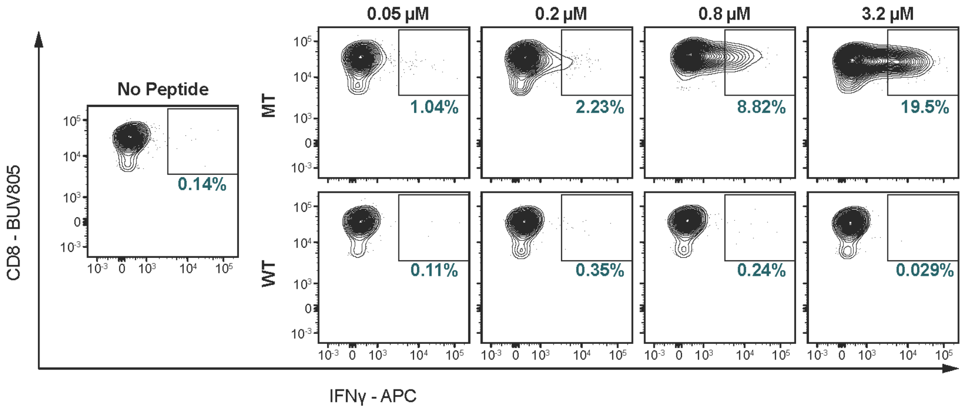

Supplementary Figure 3: Gating strategy for specificity experiment.

DP cells were co-cultured overnight with DCs presenting a range of concentrations of the relevant mutant or wild-type peptides. Upregulation of IFNγ, TNFα, and/or CD107a was measured using flow cytometry. Representative flow cytometry plots are shown for CD8⁺ IFNγ response to mutant and wild-type $PPAN_{165:P>L}$ from NAC03. Percentages are % IFNγ- secreting CD8⁺ cells out of all CD8⁺ tet⁺ T cells.

Supplementary Figure 4:

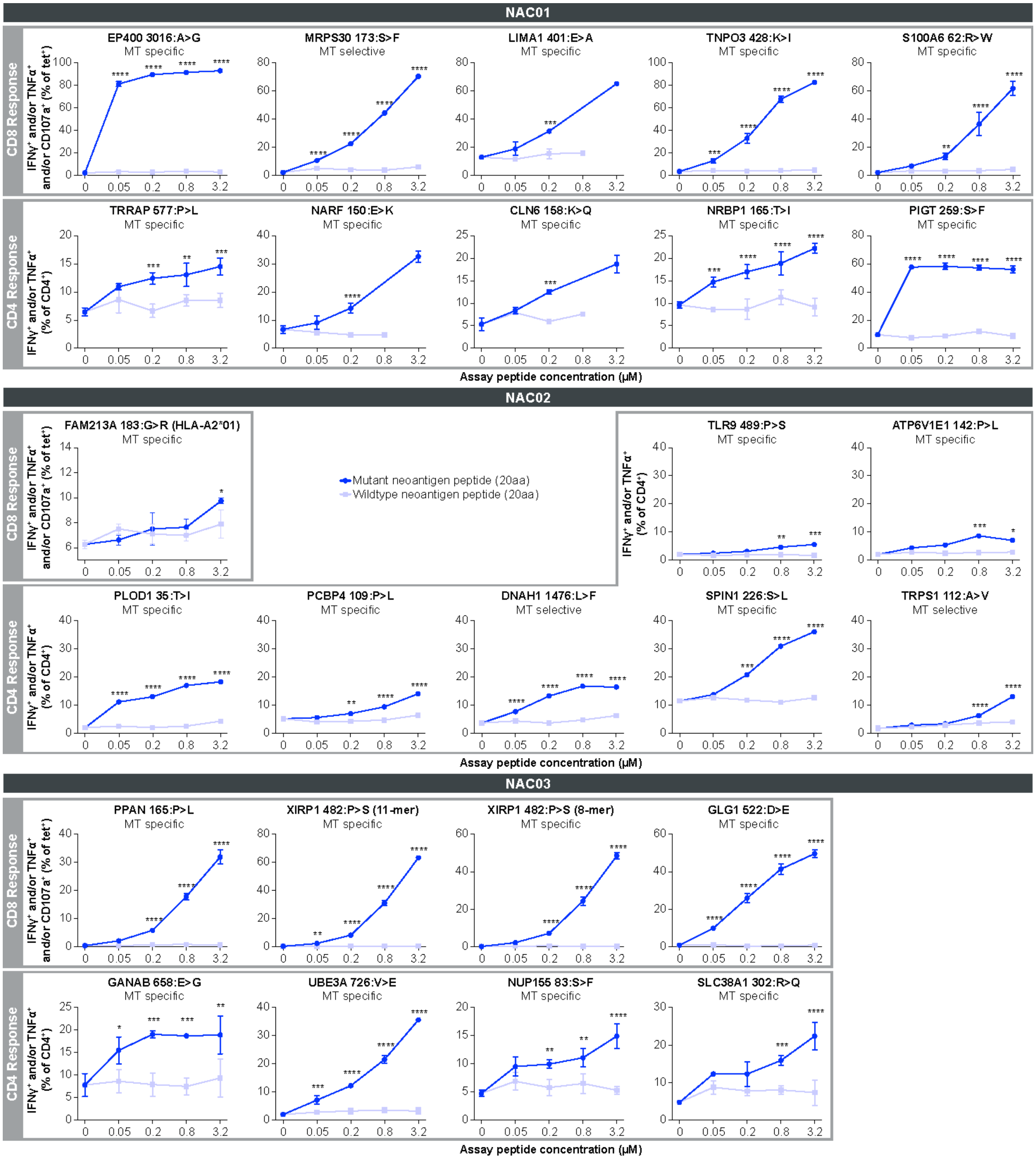

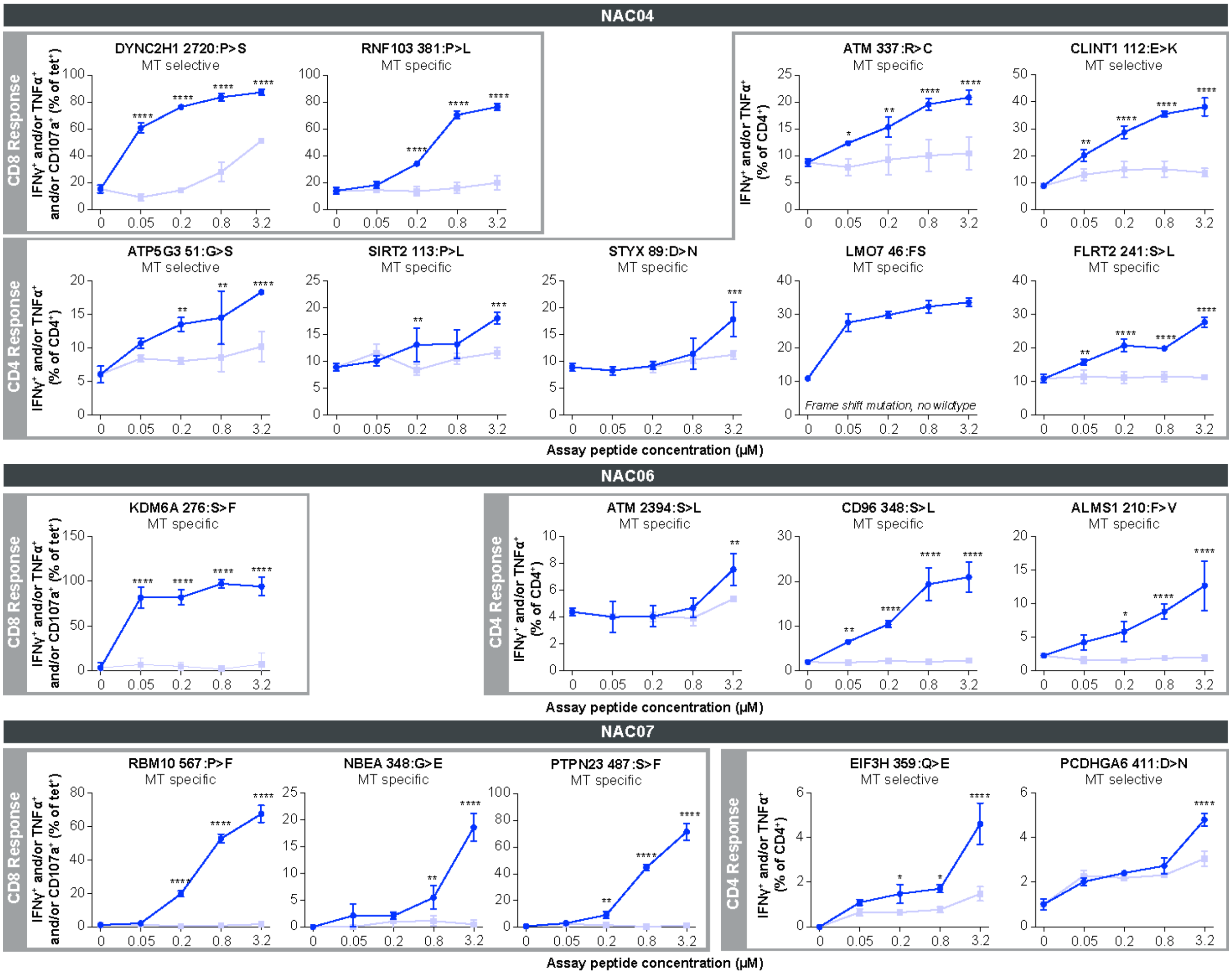

Supplementary Figure 4: Induced T cell responses are mutant-reactive.

Drug product cells from all treated patients were cocultured overnight with DCs loaded with mutant or wild-type peptide at a range of concentrations. Upregulation of IFNγ, TNFα, and/or CD107a was measured using flow cytometry. For $CD8^+$ T cells, IFNγ- and/or TNFα-secretion and/or degranulation (CD107a expression) was tested in response to recall peptide, for CD4+ T cells IFNγ- and/or TNFα-secretion. MT specific: defined as clones that showed significant reactivity to the mutant peptide over the no peptide control, and no reactivity to the wild-type peptide. MT selective: defined as clones that showed significant reactivity to the mutant peptide over the no peptide control, and reactivity to the wild-type peptide compared to the no peptide control. Data shown as mean with SD (*n*=3, technical replicates) where *p*-values of MT curve over wild-type curve were defined with a 2-way ANOVA with Sidak's multiple comparison test; *: $p \leq 0.05$; **: $p \leq 0.01$; ***: $p \leq 0.001$; ****: $p \leq 0.0001$. See Supplementary Data S2 for exact *p*-values. MT: Mutant. Abbreviations for patient IDs: NAC: neoantigen cell dose received.

Supplementary Figure 5:

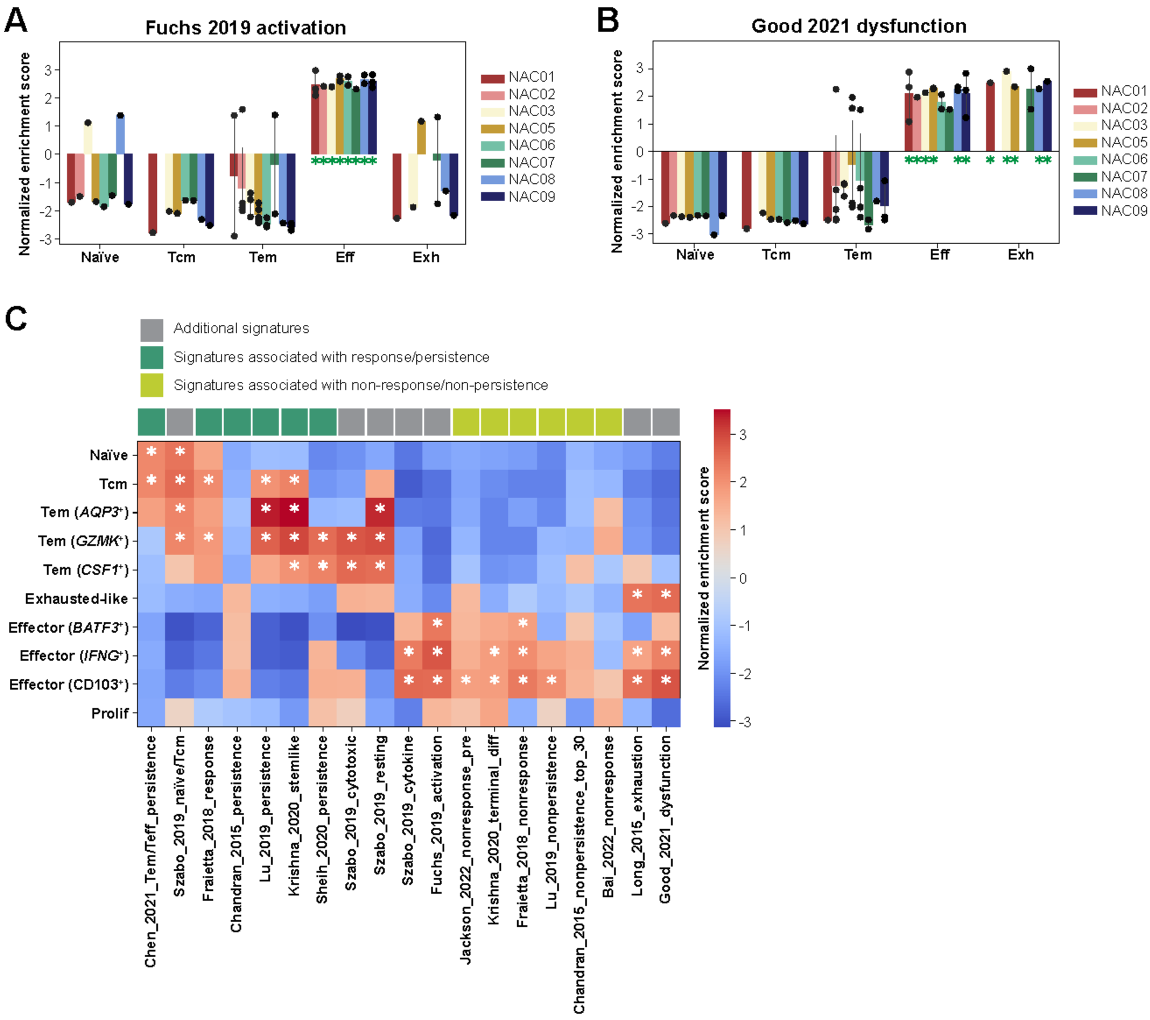

Supplementary Figure 5: Validation of cell type labels.

A, B. Gene set enrichment analysis (GSEA) by cell type for a published T cell activation signature[1] (panel A) and a published T cell dysfunction signature[2] (panel B) per patient across nine patients. Green stars: significantly enriched at false discovery rate (FDR) < 0.05. Bars represent mean and error bars SD of normalized enrichment score, in cases where a patient had multiple cell types in the indicated category (see Method section for details).

C. Representative plot of signature enrichment across 19 published gene signatures by cell type. Categorizations of signatures above the graph are from published literature. White stars: Significantly enriched at FDR < 0.05. Results shown for Patient NAC09.

References for published signatures (x-axis of heat map): Chen_2021_Tem/Teff_persistence[3], Szabo_2019_naïve/Tcm[4], Fraietta_2018_response[5], Chandran_2015_persistence[6],

Lu_2019_persistence[7], Krishna_2020_stemlike[8], Sheih_2020_persistence[9], Szabo_2019_cytotoxic[4], Szabo_2019_resting[4], Szabo_2019_cytokine[4], Fuchs_2019_activation[1], Jackson_2022_nonresponse_pre[10], Krishna_2020_terminal_diff[8], Fraietta_2018_nonresponse[5], Lu_2019_nonpersistence[7], Chandran_2015_nonpersistence_top_30[6], Bai_2022_nonresponse[11], Long_exhaustion[12], Good_2021 dysfunction[2].

Abbreviations of patient IDs: NAC: neoantigen cell dose received.

1. Fuchs, Y.F., *et al.* Gene Expression-Based Identification of Antigen-Responsive CD8(+) T Cells on a Single-Cell Level. *Front Immunol* **10**, 2568 (2019).
2. Good, C.R., *et al.* An NK-like CAR T cell transition in CAR T cell dysfunction. *Cell* **184**, 6081-6100 e6026 (2021).
3. Chen, G.M., *et al.* Integrative Bulk and Single-Cell Profiling of Premanufacture T-cell Populations Reveals Factors Mediating Long-Term Persistence of CAR T-cell Therapy. *Cancer Discov* **11**, 2186-2199 (2021).
4. Szabo, P.A., *et al.* Single-cell transcriptomics of human T cells reveals tissue and activation signatures in health and disease. *Nat Commun* **10**, 4706 (2019).
5. Fraietta, J.A., *et al.* Determinants of response and resistance to CD19 chimeric antigen receptor (CAR) T cell therapy of chronic lymphocytic leukemia. *Nat Med* **24**, 563-571 (2018).
6. Chandran, S.S., *et al.* Tumor-Specific Effector CD8+ T Cells That Can Establish Immunological Memory in Humans after Adoptive Transfer Are Marked by Expression of IL7 Receptor and c-myc. *Cancer Res* **75**, 3216-3226 (2015).
7. Lu, Y.C., *et al.* Single-Cell Transcriptome Analysis Reveals Gene Signatures Associated with T-cell Persistence Following Adoptive Cell Therapy. *Cancer Immunol Res* **7**, 1824-1836 (2019).
8. Krishna, S., *et al.* Stem-like CD8 T cells mediate response of adoptive cell immunotherapy against human cancer. *Science* **370**, 1328-1334 (2020).
9. Sheih, A., *et al.* Clonal kinetics and single-cell transcriptional profiling of CAR-T cells in patients undergoing CD19 CAR-T immunotherapy. *Nat Commun* **11**, 219 (2020).
10. Jackson, Z., *et al.* Sequential Single-Cell Transcriptional and Protein Marker Profiling Reveals TIGIT as a Marker of CD19 CAR-T Cell Dysfunction in Patients with Non-Hodgkin Lymphoma. *Cancer Discov* **12**, 1886-1903 (2022).
11. Bai, Z., *et al.* Single-cell antigen-specific landscape of CAR T infusion product identifies determinants of CD19-positive relapse in patients with ALL. *Sci Adv* **8**, eabj2820 (2022).
12. Long, A.H., *et al.* 4-1BB costimulation ameliorates T cell exhaustion induced by tonic signaling of chimeric antigen receptors. *Nat Med* **21**, 581-590 (2015).

Supplementary Figure 6

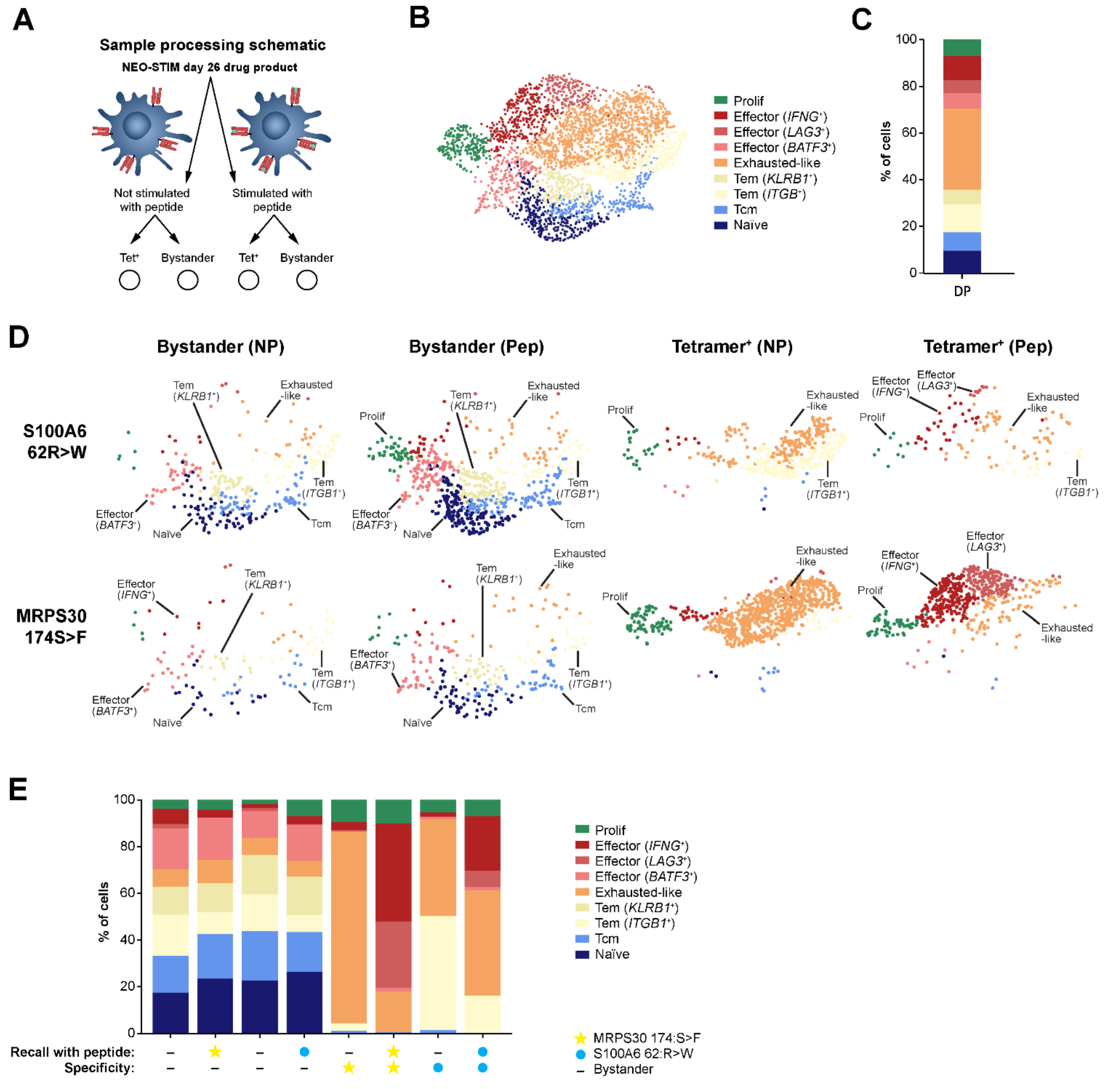

Supplementary Figure 6: Phenotype determination of drug product cells by scGEX/VDJ/CITE-seq.

This figure describes in more detail the phenotype determination approach applied in **Extended Fig. 2**. To identify the range of phenotypes for cell populations subjected to different experimental conditions, the cells from each experimental condition are sequenced; cells from all samples are analyzed jointly by uniform manifold approximation and projection (UMAP), and phenotypes are assigned to cell populations in the entire cell pool. Phenotypic distributions can then be compared directly between different samples.

- **A.** Schematic showing sequencing samples from NAC01 drug product (DP). Samples fall into four categories: not stimulated with peptide tet^{+}, not stimulated with peptide tet^{-} (bystander), stimulated tet^{+}, stimulated tet^{-} (bystander). Figure panel was created in BioRender (Gottstein, C. https://BioRender.com/e86m166 (2024)).
- **B.** UMAP of scGEX/VDJ/CITE-seq data from NAC01 DP. Cells from DP samples were integrated using multi-omics factor analysis (MOFA), projected into a shared two-dimensional space, and labeled jointly using transcriptome and surface protein markers.
- **C.** Range of phenotypes for cells from all samples of NAC01 DP.
- **D.** UMAPs showing cells from eight samples individually. Top: T cells specific for neoantigen 1 ($S100A6_{62R>W}$). Bottom: T cells specific for neoantigen 2 ($MRSP30_{174S>F}$). Labels are shown in each panel for cell types present at frequency of >5%.
- **E.** Range of phenotypes for eight individual experimental samples from NAC01 DP. This is the cell type distribution shown in **Extended Fig. 2b**, lower panel.

Abbreviations of patient IDs: NAC: neoantigen cell dose received.

Supplementary Figure 7:

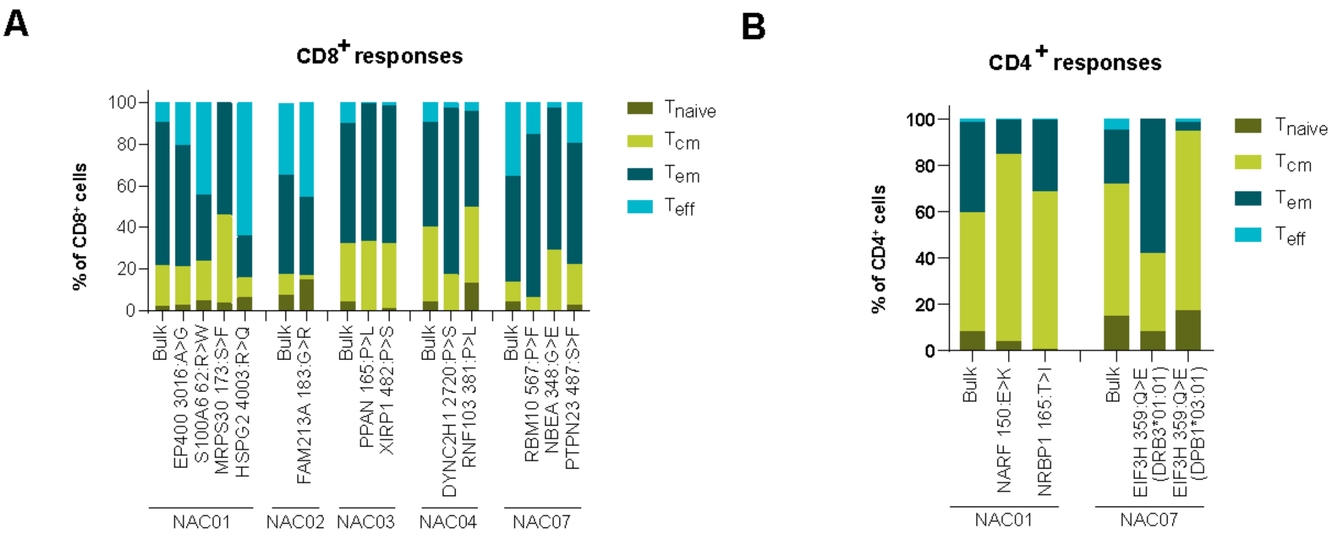

Supplementary Figure 7: Phenotype determination of neoantigen-specific T cells post-infusion.

- **A.** Frequency of $CD8^+$ Tnaïve, Tcm, Tem and Teff, defined using CD45RA and CCR7 across patients and individual responses identified by tetramer staining.
- **B.** Frequency of $CD4^+$ Tnaïve, Tcm, Tem and Teff, defined using CD45RA and CCR7 across patients and individual responses identified by tetramer staining.

Abbreviations for patient IDs: NAC: <u>n</u>eo<u>a</u>ntigen <u>c</u>ell dose received.

Supplementary Figure 8:

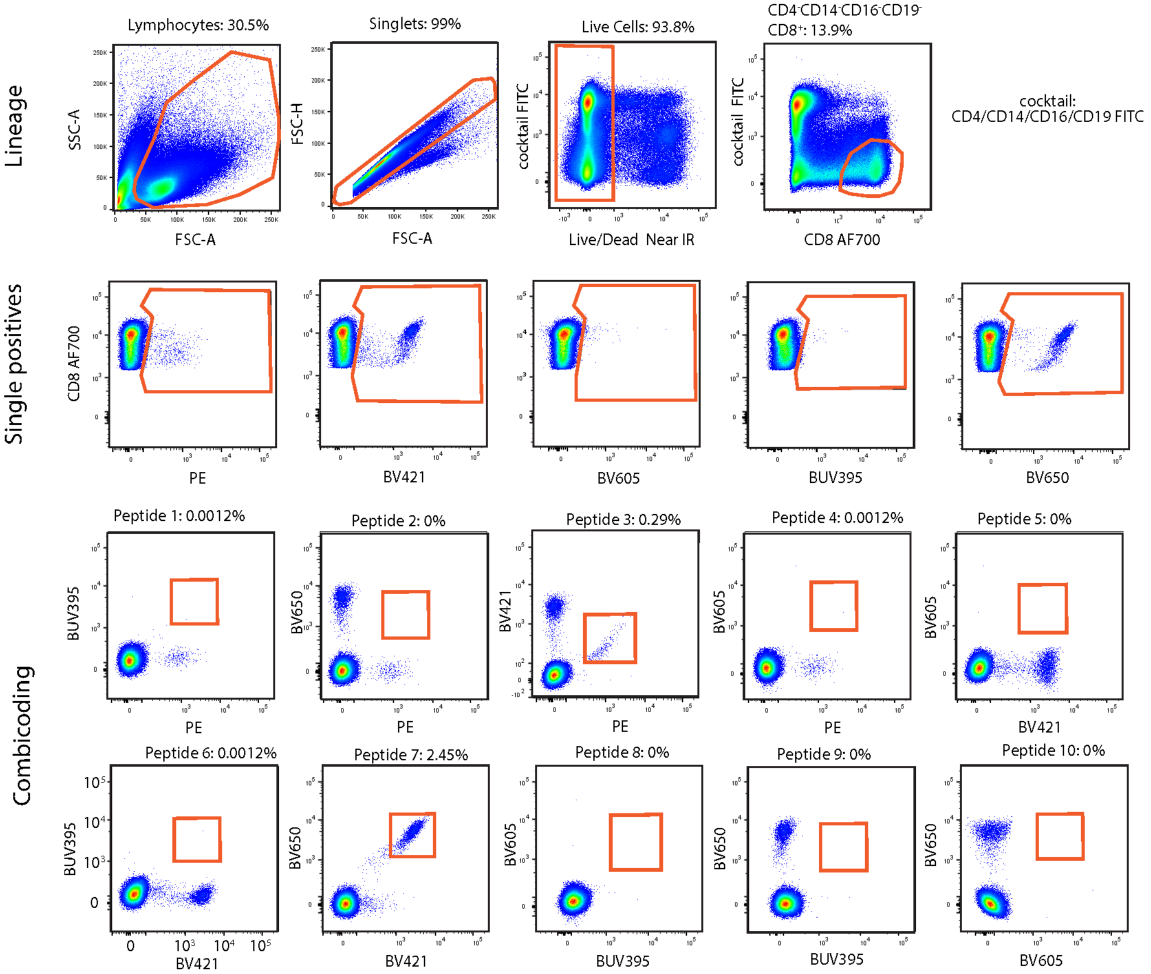

Supplementary Figure 8: Example for Flow Cytometry gating strategy.

This example illustrates the gating flow for tetramer staining of CD8+ cells from patient NAC01.

Lineage: Forward scatter and side scatter gates are applied to filter for singlets of the target cell population PBMC. Dead cells are excluded with a live/dead marker. Cells are then gated for positive CD8 staining and negative CD4/CD14/CD16 and CD19 staining.

Single positives: CD8^{+} cells stained with the single fluorophores, which are used for the tetramer labelling, are gated.

Combicoding: Peptide – MHC complexes are labelled with two different fluorophores to make tetramers. Double positivity for both fluorophores that have been assigned to a specific peptide in the combinatorial coding process are considered positive for the respective neoantigen.

Example: 2.45% of CD8^{+} cells are specific for peptide #7.

Supplementary Data Tables

Supplementary Data Table 1: Overall study-related AEs.

System organ class	Preferred term	Dose Level 1 (n=3) n (%)	Dose Level 2 (n=6) n (%)	Total (n=9) n (%)
**Any related AE**		**3 (100)**	**6 (100)**	**9 (100)**
**Blood and lymphatic disorders**		**3 (100)**	**5 (83)**	**8 (89)**
	Anemia	3 (100)	5 (83)	8 (89)
**Cardiac disorders**		**1 (33)**	**0 (0)**	**1 (11)**
	Conduction disorders	1 (33)	0 (0)	1 (11)
**Gastrointestinal disorders**		**3 (100)**	**6 (100)**	**9 (100)**
	Constipation	2 (67)	3 (50)	5 (56)
	Diarrhea	0 (0)	1 (17)	1 (11)
	Nausea	2 (67)	6 (100)	8 (89)
**General disorders and administration site conditions**		**2 (67)**	**4 (67)**	**6 (67)**
	Fatigue	1 (33)	4 (67)	5 (56)
	Edema peripheral	1 (33)	0 (0)	1 (11)
	Pyrexia	1 (33)	2 (33)	3 (33)
**Infections and infestations**		**2 (67)**	**3 (50)**	**5 (56)**
	Candida infection	1 (33)	0 (0)	1 (11)
	Device-related infection	0 (0)	3 (50)	3 (33)
	Herpes simplex reactivation	0 (0)	1 (17)	1 (11)
	Skin infection	1 (33)	0 (0)	1 (11)
**Investigations**		**3 (100)**	**6 (100)**	**9 (100)**
	Blood creatinine increased	0 (0)	1 (17)	1 (11)
	Blood lactate dehydrogenase increased	1 (33)	0 (0)	1 (11)
	Lymphocyte count decreased	3 (100)	6 (100)	9 (100)
	Neutrophil count decreased	3 (100)	6 (100)	9 (100)
	Platelet count decreased	1 (33)	1 (17)	2 (22)
	Weight increased	0 (0)	5 (83)	5 (56)
	White blood cell count decreased	3 (100)	6 (100)	9 (100)
**Metabolism and nutrition disorders**		**0 (0)**	**2 (33)**	**2 (22)**
	Decreased appetite	0 (0)	2 (33)	2 (22)
**Musculoskeletal and connective tissue disorders**		**2 (67)**	**0 (0)**	**2 (22)**
	Back pain	2 (67)	0 (0)	2 (22)
**Nervous system disorders**		**2 (67)**	**3 (50)**	**5 (56)**
	Dysgeusia	1 (33)	2 (33)	3 (33)
	Headache	0 (0)	2 (33)	2 (22)
	Paresthesia	1 (33)	1 (17)	2 (22)

Presyncope	1 (33)	0 (0)	1 (11)
**Respiratory, thoracic and mediastinal disorders**	**1 (33)**	**0 (0)**	**1 (11)**
Cough	1 (33)	0 (0)	1 (11)
**Skin and subcutaneous tissue disorders**	**3 (100)**	**4 (67)**	**7 (78)**
Alopecia	1 (33)	4 (67)	5 (56)
Erythema	1 (33)	0 (0)	1 (11)
Pruritus	1 (33)	0 (0)	1 (11)
Rash maculo-papular	0 (0)	2 (33)	2 (22)
Scar pain	1 (33)	0 (0)	1 (11)
**Vascular disorders**	**0 (0)**	**1 (17)**	**1 (11)**
Flushing	0 (0)	1 (17)	1 (11)

Overall study-related adverse events (AEs) by system organ class (SOC) term (bold), preferred term (PT), incidence and grade. Assignment to dose levels per intent to treat. See Extended Table 2 for actual doses received. Note that headers are also in bold.

Supplementary Data Table 2: Planned changes during trial conduct.

Time	Change	Reason
CTP04 (Jan 26, 2021)	Patients may receive melanoma directed therapy prior to BNT221 and will be fully evaluated	Opportunity for treatment intervention during BNT221 manufacturing period, for patients with advanced and metastatic melanoma nonresponsive to PD-1 and CTLA-4-directed therapies
CTP04 (Jan 26, 2021)	Patients who have disease progression or otherwise lack of clinical benefit (per study investigator) on PD-1 inhibitors are eligible, as are those with prior treatment discontinuation due to ICB-related toxicities	Expand the patient population who may receive BNT221 for their unresectable or metastatic melanoma
CTP04 (Jan 26, 2021)	Study population: BRAF mutant patients must have received BRAF inhibitor or BRAF/MEK inhibitor combination therapy prior to study entry, unless such therapy is precluded per the study investigator	Ensure BRAF mutant patients have received standard of care treatments for their advanced or metastatic disease prior to phase 1 study
CTP07 (Aug 16, 2022)	Introduce two new subgroups in Part 1:<br>1. BNT221 treatment followed by PD-1 inhibitor<br>2. BNT221 treatment co-administered with IL-2	Following determination of BNT221 monotherapy MTD, combination regimens were added to evaluate BNT221 with immunodulator therapies

Supplementary Data Files

Supplementary Data S1: List of neoepitopes tested (Excel)

See separate excel file

Supplementary Data S2: Mutant specificity calculations (*p*-values, Excel).

See separate excel file

Supplementary Data S3: Characterization of T cell receptors (Excel)

See separate excel file

Supplementary Data S4: Antibody List and Flow panel

See separate excel file
