# Supplementary material for: Personalized, autologous neoantigen-specific T cell therapy in metastatic melanoma: a phase 1 trial
Source: Nat Med. 2025 Jan 3;31(3):881–93. doi: 10.1038/s41591-024-03418-4 (PMC11922764; doi:10.1038/s41591-024-03418-4)
Supplement: Supplementary file 2 — Reporting Summary [file 41591_2024_3418_MOESM2_ESM.pdf]

nature portfolio

Corresponding author(s): Marit M van Buuren, John B Haanen

Last updated by author(s): 11/06/2024

Reporting Summary

Nature Portfolio wishes to improve the reproducibility of the work that we publish. This form provides structure for consistency and transparency in reporting. For further information on Nature Portfolio policies, see our Editorial Policies and the Editorial Policy Checklist.

Statistics

For all statistical analyses, confirm that the following items are present in the figure legend, table legend, main text, or Methods section.

n/a	Confirmed	
☐	☒	The exact sample size (*n*) for each experimental group/condition, given as a discrete number and unit of measurement
☐	☒	A statement on whether measurements were taken from distinct samples or whether the same sample was measured repeatedly
☐	☒	The statistical test(s) used AND whether they are one- or two-sided<br>*Only common tests should be described solely by name; describe more complex techniques in the Methods section.*
☒	☐	A description of all covariates tested
☒	☐	A description of any assumptions or corrections, such as tests of normality and adjustment for multiple comparisons
☐	☒	A full description of the statistical parameters including central tendency (e.g. means) or other basic estimates (e.g. regression coefficient) AND variation (e.g. standard deviation) or associated estimates of uncertainty (e.g. confidence intervals)
☐	☒	For null hypothesis testing, the test statistic (e.g. *F*, *t*, *r*) with confidence intervals, effect sizes, degrees of freedom and *P* value noted<br>*Give P values as exact values whenever suitable.*
☒	☐	For Bayesian analysis, information on the choice of priors and Markov chain Monte Carlo settings
☒	☐	For hierarchical and complex designs, identification of the appropriate level for tests and full reporting of outcomes
☒	☐	Estimates of effect sizes (e.g. Cohen's *d*, Pearson's *r*), indicating how they were calculated

Our web collection on statistics for biologists contains articles on many of the points above.

Software and code

Policy information about availability of computer code

Data collection	No code was used.
Data analysis	Statistical analysis of clinical data: SAS Enterpride Guide (version 8.4). IHC analysis: Image J (version 1.53k); building TCR vectors: SnapGene (versions 4.2, 4.3, 5.0); alignment and mutational calling, and neoantigen peptide selection; proprietary custom bioinformatics analysis; gene set enrichment analysis: GSEAPy, version 1.1.3; TCR repertoire generation: MiXCR version 4.3.2; alignment of raw scGEX/VDJ/CITE-seq data: Cell Ranger version 6.0.1 (10x genomics); flow cytometry: FlowJo, versions 08, 09, and 10; all other analyses: Python language (version 3.9.15), or GraphPad Prism (version 7.01).

For manuscripts utilizing custom algorithms or software that are central to the research but not yet described in published literature, software must be made available to editors and reviewers. We strongly encourage code deposition in a community repository (e.g. GitHub). See the Nature Portfolio guidelines for submitting code & software for further information.

Data

Policy information about availability of data

All manuscripts must include a data availability statement. This statement should provide the following information, where applicable:

- Accession codes, unique identifiers, or web links for publicly available datasets
- A description of any restrictions on data availability
- For clinical datasets or third party data, please ensure that the statement adheres to our policy

This trial is currently ongoing. Upon completion of this clinical trial, summary-level results will be made public and shared in line with clinical data-sharing guidelines. Requests for access to aggregated clinical data will be reviewed and approved by the Safety Review Committee on the basis of scientific merit. The datasets generated during the current study and the custom bioinformatics analysis are not publicly available due to proprietary considerations beyond the data that were made available here. All data provided are anonymized to respect the privacy of patients who have participated in the trial, in line with applicable laws and regulations. Data requests pertaining to the manuscript may be made to the corresponding authors (M.M.v.B., marit.vanbuuren@biontech.us and J.B.H., j.haanen@nki.nl). Requests will be processed within 16 weeks. NEO-STIM patents are pending in various countries (WO2020227546A1, WO2023064930A1).

Datasets generated in the study were: patient derived single cell GEX/CITE/VDJ and bulk TCR datasets generated at multiple timepoints.
Public datasets and databases used in this study were:
- GRCh38-2020-A genome (https://www.10xgenomics.com/support/software/cell-ranger/downloads#reference-downloads);
- vdj_GRCh38_alts_ensembl-7.1.0 assembly (https://www.10xgenomics.com/support/software/cell-ranger/downloads/eula?closeUrl=%2Fsupport%2Fsoftware%2Fcell-ranger&lastTouchOfferName=Cell%20Ranger&lastTouchOfferType=Software%20Download&product=chromium&redirectUrl=%2Fsupport%2Fsoftware%2Fcell-ranger%2Fdownloads%23reference-downloads);
- 10x genomics Immunological panel 1056 genes (https://www.10xgenomics.com/support/single-cell-gene-expression/documentation/steps/targeted-gene-expression/human-immunology-panel);
- Activation score (Fuchs et al. 2019, Table S5 (tab 'TOP50 separators_expression', doi: 10.3389/fimmu.2019.02568, https://www.frontiersin.org/articles/10.3389/fimmu.2019.02568/full#supplementary-material);
- Dysfunction score (Good et al. 2021 (doi: 10.1016/j.cell.2021.11.016) ;
- mSigDB Hallmark collection, Reactome, KEGG, BioCarta and the Pathway interaction database were downloaded from https://www.gsea-msigdb.org/gsea/msigdb and some curation was performed for this manuscript.

Human research participants

Policy information about studies involving human research participants and Sex and Gender in Research.

Reporting on sex and gender	Sex was ascertained at screening by the investigator. Gender was not reported. Given the limited study size, outcomes were not formally analyzed according to the sex distribution.
Population characteristics	Information on age, sex, ECOG, performance status, cancer type and stage and pretreatment was collected at screening. All patients had prior treatment with PD-1 and CTLA4-directed therapies and had non-response or intolerance to these therapies prior to study entry. All patients had stage 4 melanoma at study entry. Of the above parameters, number of pretreatment cancer regimens, ECOG performance status, age, and sex are potential covariates. No formal subanalyses based on population characteristics were conducted due to the limited sample in this phase 1 trial.
Recruitment	Participants were recruited by the clinical trial center per eligibility criteria outlined in the manuscript. Prior to any non-routine analysis, sampling or intervention, each patient was fully informed about the study, and signed an informed consent form. Patients were not compensated for participating in the study.
Ethics oversight	The study was approved on April 24, 2020 by the Central Committee on Research Involving Human Subjects (CCMO), approval no. NL72301.000.19.

Note that full information on the approval of the study protocol must also be provided in the manuscript.

Field-specific reporting

Please select the one below that is the best fit for your research. If you are not sure, read the appropriate sections before making your selection.

☒ Life sciences ☐ Behavioural & social sciences ☐ Ecological, evolutionary & environmental sciences

For a reference copy of the document with all sections, see nature.com/documents/nr-reporting-summary-flat.pdf

Life sciences study design

All studies must disclose on these points even when the disclosure is negative.

Sample size	The sample size was determined according to the 3+3 dose escalation method.

Data exclusions	No data was excluded. Due to sample availability translational analyses were not always performed on all patients.
Replication	Clinical samples were analyzed longitudinally (single samples). Translational analyses were run with two to four technical replicates, where applicable (Fig. 3C-F,H; Suppl Fig 4) and data is represented as mean and standard deviation. Some experiments were repeated and replication was successful (Fig 3C CD8+, Figure 4A,B,C; Suppl Fig 2).
Randomization	This is not a randomized study. Allocation was per 3+3 dose escalation design principles.
Blinding	N/A, this was an open label non blinded study.

Reporting for specific materials, systems and methods

We require information from authors about some types of materials, experimental systems and methods used in many studies. Here, indicate whether each material, system or method listed is relevant to your study. If you are not sure if a list item applies to your research, read the appropriate section before selecting a response.

Materials & experimental systems

n/a	Involved in the study	
☐	☒	Antibodies
☐	☒	Eukaryotic cell lines
☒	☐	Palaeontology and archaeology
☒	☐	Animals and other organisms
☐	☒	Clinical data
☒	☐	Dual use research of concern

Methods

n/a	Involved in the study	
☒	☐	ChIP-seq
☐	☒	Flow cytometry
☒	☐	MRI-based neuroimaging

Antibodies

Antibodies used	A list of all antibodies and dilutions is provided in Supplemental Dataset S4.
Validation	Antibody validation data are available from the manufacturers' websites. Antibody concentrations were titrated and verified for the application. Validation methods used by the manufacturer may be found in Supplemental Dataset S4.

Eukaryotic cell lines

Policy information about cell lines and Sex and Gender in Research

Cell line source(s)	Jurkat and A375 cells were from ATCC. Further sub cell lines were created in house.
Authentication	Cell line authentication was performed by the manufacturer via STR profiling. No further authentication has been performed.
Mycoplasma contamination	Not all cell lines / sub cell lines were tested for mycoplasma contamination. When tested, they were negative for mycoplasma contamination.
Commonly misidentified lines (See ICLAC register)	The cell lines used are not listed on the ICLAC register of misidentified cell lines (version 13), nor in the list of cross-contaminated cell lines (Capes-Davis et al. Int J Cancer 127:108, 2010).

Clinical data

Policy information about clinical studies

All manuscripts should comply with the ICMJE guidelines for publication of clinical research and a completed CONSORT checklist must be included with all submissions.

Clinical trial registration	NCT04625205
Study protocol	The full clinical trial protocol has been submitted for review. For the publication, full eligibility criteria, as well as detailed study design are described in the Online methods section. The full trial protocol, beyond what is shared in the manuscript, is proprietary.
Data collection	The study has been performed at the Netherlands Cancer Institute in Amsterdam. Patients for the monotherapy arm, which is reported here, were enrolled between December 2020 and October 2022. The data cutoff of December 15, 2023 was used for clinical data. One datapoint (lymphocyte count) was queried and updated to the database in May 2024.
Outcomes	Primary objectives for part 1 were evaluation of safety and identification of highest tolerable dose. Endpoints were Adverse Events (AEs), serious AEs (SAEs) and AEs leading to treatment discontinuation, as well as changes in laboratory values, physical examination findings. The secondary objective of the study is evaluation of antitumor activity and endpoints include overall response rate based on RECIST 1.1., duration of response (DOR), clinical benefit rate (CBR) and progression free survival (PFS).

Exploratory objectives included the characterization of the immune response before, during and after treatment and the characterization of the clonal expansion, persistence and phenotype of the transferred cells.

Flow Cytometry

Plots

Confirm that:

☒ The axis labels state the marker and fluorochrome used (e.g. CD4-FITC).

☒ The axis scales are clearly visible. Include numbers along axes only for bottom left plot of group (a 'group' is an analysis of identical markers).

☒ All plots are contour plots with outliers or pseudocolor plots.

☒ A numerical value for number of cells or percentage (with statistics) is provided.

Methodology

Sample preparation	Blood draws or leukapheresis were performed to collect PBMCs from the patients. PBMCs were isolated through Ficoll or washing steps. PBMCs or manufactured Drug Product were frozen in DMSO-containing media. Materials were thawed and used in downstream assays.
Instrument	Instruments used for analysis were BD LSR Fortessa or BD FACSymphony A5. BD FACSAria was used for sorting.
Software	For flow cytometry analysis the software FlowJo, versions 08, 09, and 10 were used.
Cell population abundance	Single cell sorting was performed on BD FACSAria and purity of sorted populations was promoted by setting conservative gates for lineage markers/pMHC multimers and utilizing the "purity sort mode" to restrict the presence of cells in adjacent droplets during sorting.<br>Approximate processed cell number for each different condition was as follows:<br>- PBMC CD8+ bulk: ~6,000 cells<br>- DP CD8+ pMHC- bystanders: ~2,000 cells<br>- DP CD8+ pMHC+: ~50-2,000 cells<br>Purity of samples post-sort was evaluated post library generation by cell type categorization utilizing transcriptome and targeted proteome profiles, and final analysis only included CD8+ cells.
Gating strategy	Gating strategies for the different experiments are described in the Online Methods. An example for gating strategy is shown in Supplementary Figure 8.

☒ Tick this box to confirm that a figure exemplifying the gating strategy is provided in the Supplementary Information.
